# Study on regional differences and causes of emergency logistics responsiveness under the impact of public emergencies—findings from Chinese samples

**DOI:** 10.3389/fpubh.2024.1508467

**Published:** 2024-12-13

**Authors:** Heng Chen, Yuan Guo, Xianglong Lin, Xianchao Qi

**Affiliations:** School of Management, Xi’an Polytechnic University, Xi’an, China

**Keywords:** impact of public emergencies, emergency logistics responsiveness, causes of regional differences, Chinese sample, the entropy-weighted TOPSIS method

## Abstract

**Introduction:**

In recent years, the frequency and intensity of natural disasters and public emergencies around the world have been steadily increasing. Emergency logistics plays an irreplaceable role in providing rapid material and service support in the aftermath of disasters. Therefore, systematically analyzing the differences in emergency logistics responsiveness across various regions of China and understanding their underlying causes are of great significance for optimizing emergency logistics systems and improving disaster responsiveness.

**Methods:**

In order to identify the regional differences and causes of China’s emergency logistics responsiveness under the impact of public emergencies, this paper pioneered the development of an index system for evaluating emergency logistics responsiveness. Based on the panel data of 30 provinces in China from 2012 to 2021, this paper used the entropy-weighted TOPSIS method to quantify the emergency logistics responsiveness of various regions in China. In addition, this study uses a panel quantile regression model to evaluate the differences in emergency logistics responsiveness in various regions of China under the impact of public emergencies, and the causes of regional differences are explored.

**Results:**

The research results show that: (1) China’s emergency logistics responsiveness is upward, but the regional differences are expanding. (2) Compared with the central and eastern regions, the western region’s emergency logistics responsiveness has continuously improved due to the expanding scale of public emergencies. However, the emergency logistics responsiveness of the eastern region has constantly been reduced due to the impact of public emergencies. In contrast, the central region has been reduced first and then improved. (3) The level of emergency logistics technology cannot effectively promote emergency logistics responsiveness. Under the impact of public emergencies, the labor input of the logistics industry cannot effectively meet the needs of emergency logistics activities. The administrative command method and the level of marketization inhibit emergency logistics responsiveness. The improvement of the social labor input level, urbanization level, logistics development level, and digitalization level can effectively promote emergency logistics responsiveness.

**Discussion:**

The above results show that China should pay attention to regional differences. Each region should rely on the existing logistics system and plan and build emergency logistics hubs according to the characteristics of emergencies in each region. Continue to strengthen regional exchanges and cooperation to narrow the gap in regional emergency logistics responsiveness. At the same time, this paper plays a driving role in China’s joint emergency logistics rescue cooperation with other countries worldwide.

## 1 Introduction

In recent years, the frequency and intensity of natural disasters and other unforeseen public emergencies have gradually increased worldwide and even become normalized (1). As one of the countries with the most severe natural disasters in the world, China has the characteristics of many types of disasters: wide distribution, high frequency of occurrence, and heavy losses (2). According to the “2021 Global Natural Disaster Assessment Report,” China experienced 21 major natural disasters in 2021, causing 107 million people to be affected, 765 people to die, 102 people to go missing, and direct economic losses of up to 334.02 billion yuan. However, the severe casualties and financial losses caused by public emergencies have also challenged China’s regional disaster management and emergency response systems (3). Emergency logistics, as the material basis of the emergency response system for emergencies, can provide critical support for material and personnel needs in emergencies such as natural disasters (4). Effective emergency logistics plays a vital role in reducing the impact of disasters and ensuring the timely provision of basic materials and services to disaster-stricken areas (5). Therefore, it has received significant attention from the international academic community, governments, and the public.

Emergency logistics occupies an important position in the national emergency management system. It is a crucial link to ensure the rapid and efficient deployment and supply of critical materials in emergencies and to maintain social stability and people’s safety. Therefore, emergency logistics research has received attention from academia, government, and society. Firstly, although existing studies have explored the definition, classification, and potential economic impact of public emergencies, most use qualitative analysis methods. There is a lack of quantitative evaluation of the impact of public emergencies. In addition, although some studies attempt to evaluate emergency logistics responsiveness, the evaluation methods usually rely on subjective assignment or mathematical models. This may affect the accuracy and reliability of the evaluation results. In addition, the indexes used are relatively single and cannot systematically and comprehensively reflect the responsiveness of regional emergency logistics. Finally, regarding the regional differences in China’s emergency logistics responsiveness and causes, existing research has mainly explored the regional differences in the development of the logistics industry. Due to differences in the operation, organization, and participation methods of social and emergency logistics, there will also be regional differences in different regions’ emergency logistics responsiveness. Such differences may lead to constraints on cross-regional emergency logistics joint action capacities. However, existing research pays less attention to the regional differences in emergency logistics responsiveness and the specific causes of the differences, which is not conducive to the scientific and reasonable construction of an emergency logistics responsiveness system.

Therefore, the study goals of the paper are: (1) Combining the constituent factors of emergency logistics responsiveness, a comprehensive index system for evaluating emergency logistics responsiveness is constructed to measure the emergency logistics responsiveness of different regions in China. (2) Study the regional differences in China’s emergency logistics responsiveness under the impact of public emergencies. (3) Empirical methods will be used to identify the causes of differences in China’s regional emergency logistics responsiveness.

The rest of this paper is organized as follows: Section 2 conducts a literature review. Section 3 is the measurement of China’s regional emergency logistics responsiveness and the analysis of regional differences. Section 4 examines the causes of the differences in China’s regional emergency logistics responsiveness. Section 5 is a discussion section, including the innovation and significance of this study. Section 6 is the conclusion and implications.

## 2 Literature review

In combination with the purpose and theme of this study and combination with relevant domestic and foreign literature, existing research mainly focuses on three main aspects: the definition, classification, and impact of public emergencies, emergency logistics responsiveness assessment, and the differences in regional logistics industry development, as follows:

### 2.1 Research on the definition, classification, and impact of public emergencies

As a key factor affecting economic progress and social stability, public emergencies have caused in-depth discussions in the academic community on their definition, classification, and impact effects. For example, Qi et al. (6) studied that the definition of sudden public events refers to the sudden outbreak of events that jeopardize people’s lives, properties, social security, and stability, which has the characteristics of destructiveness, uncertainty, comprehensiveness, sociality, suddenness, and urgency, etc. Jiang et al. (7) identified the characteristics of large-scale natural disasters by combing through the literature. Xue et al. (8) classified public emergencies into five categories: natural disasters, accidental disasters, public health emergencies, social security emergencies, and economic crises. Studies on the impact of public emergencies have mainly focused on the impact of public health events and natural disasters on socioeconomic growth. Hadian et al. (9) included studies that explored the direct and indirect costs of nine different acute respiratory diseases and the broader economic impact. Ozili et al. (10) empirically studied the impact of COVID-19 cases and COVID-19 deaths on global macroeconomic performance from 2020 to 2021. Mofijur et al. (11) comprehensively analyzed the impact of the COVID-19 epidemic on the ecological field, energy sector, society and economy and investigated global preventive measures taken to reduce the spread of COVID-19. Huang et al. (12) evaluated the post-disaster economic impact of the 2008 Sichuan earthquake and its regional and industry spillover effects based on China’s multi-regional input–output tables.

### 2.2 Research on emergency logistics responsiveness assessment

As an essential part of emergency management, emergency logistics responsiveness runs through all stages of emergency response and provides strong support for responding to public emergencies. Therefore, many scholars have researched evaluating emergency logistics responsiveness. For example, Huang et al. (13) studied grain’s emergency logistics supply capacity, established a corresponding evaluation index system, used the intuitive fuzzy analytic hierarchy process for a comprehensive evaluation, and proposed a method for evaluating grain’s emergency logistics supply capacity in response to natural disasters. Yang et al. (14) applied the analytic hierarchy process and gray system theory to establish a gray comprehensive evaluation model for the port emergency logistics distribution system. Xu et al. (15) constructed an evaluation index system for emergency logistics support capacity and an evaluation model based on triangular fuzzy entropy and Choquet integral. Zhang et al. (16) established an emergency logistics capacity evaluation model based on the BP neural network and applied the evaluation model to the COVID-19 epidemic. Acimovic et al. (17) generated new emergency logistics indexes based on a stochastic optimization model to meet critical needs after sudden disasters. It can be seen that most of the existing studies use the fuzzy analytic hierarchy process and cloud model, and the determination of index weights is mainly achieved through subjective weighting methods such as the analytic hierarchy process. Some studies on emergency logistics capacity evaluation are shown in Table 1.

**Table 1:** Compilation of research related to emergency logistics capacity assessment.

Author	Study subject	Evaluation method
Huang et al.	Food emergency logistics	Intuitive fuzzy AHP
Yang et al.	Port emergency logistics	Analytical hierarchy process
Xu et al.	Emergency logistics during natural disasters	Triangular fuzzy entropy and Choquet integral
Zhang et al.	Emergency logistics during the COVID-19 pandemic	BP neural network
Acimovic et al.	Disaster emergency logistics	Stochastic optimization model

### 2.3 Differences in regional logistics industry development

Studying the differences in regional logistics development has essential academic and practical significance for optimizing policy formulation and improving transportation efficiency. Therefore, many scholars have researched the differences in regional logistics development in response to logistics development in different regions. Bilovodska et al. (18) used the Logistics Performance Index (LPI) to analyze Ukraine’s logistics potential, compare it with other countries, and propose a development procedure for corporate distribution systems. Xiao et al. (19) used the spatial distribution of regional logistics energy utilization and carbon emissions as two indexes of green logistics to study the regional differences and changes in spatiotemporal logistics energy efficiency. Ran et al. (20) collected relevant data on the development of the logistics industry in eight economic regions in China from 2009 to 2018. The DEA model was used to calculate the logistics efficiency of 31 provinces and cities in these eight economic regions. Takele et al. (21) used the international trade gravity model of Heckman maximum likelihood regression to explore the impact of trade logistics performance on intra-regional trade in Africa. Liu et al. (22) found that in the post-epidemic era, China’s logistics industry will develop rapidly, and major changes will occur in five aspects: logistics demand, logistics supply, logistics infrastructure, logistics informatization, and logistics industry development.

### 2.4 Research gaps

By reviewing and integrating existing academic achievements. In terms of the definition, classification, and impact of public emergencies. However, existing studies have conducted preliminary discussions on the definition and classification of public emergencies, providing some inspiration for this study. However, most of these studies remain at the qualitative level and lack in-depth quantitative analysis of the impact of public emergencies. In terms of the evaluation of emergency logistics responsiveness, the indexes used in existing research are relatively simple and cannot systematically and comprehensively reflect the regional emergency logistics responsiveness. In addition, the evaluation methods currently used tend to be subjective assignment methods or mathematical models, which may lead to subjective bias in the evaluation process and affect the accuracy and reliability of the evaluation results. In terms of the differences in the development of regional logistics industries, some scholars mainly focus on the development of regional logistics industries. However, the development of logistics industries does not mean that emergency logistics responsiveness is equally improved. Due to the differences in the operation mode, organization mode, and participation mode of general logistics and emergency logistics, there will be regional differences in the emergency logistics responsiveness of different regions. Such differences may lead to constraints in cross-regional emergency logistics joint action responsiveness. However, existing research needs to pay more attention to regional emergency logistics responsiveness differences and their causes.

Based on this, this paper has made the following extensions based on existing domestic and foreign research results: (1) A compositive index framework is established to assess the emergency logistics responsiveness of various regions in China. The framework integrates multiple key factors affecting emergency logistics and applies the entropy weight TOPSIS method for evaluation. (2) The Dagum Gini coefficient is used to explore the regional differences and sources of differences in China’s regional emergency logistics responsiveness, and the panel quantile regression method is constructed to analyze the regional differences in China’s emergency logistics responsiveness under the impact of public emergencies. (3) An empirical method is used to identify the causes of regional differences in China’s regional emergency logistics responsiveness. The gaps between this research and existing research are shown in Table 2.

**Table 2:** Research gaps.

Research field	Existing research	This research
Research on the definition, classification, and impact of public emergencies	Existing studies have conducted preliminary discussions on the definition and classification of public emergencies. However, most of these studies remain at the qualitative level and lack in-depth quantitative analysis of the impact of public emergencies.	This research conducts a quantitative study on the impact of public emergencies. A panel quantile regression method was constructed to analyze the regional differences in China’s emergency logistics responsiveness under the impact of public emergencies.
Research on emergency logistics responsiveness assessment	In the evaluation of emergency logistics responsiveness, the methods currently used in research tend to be subjective assignment methods or mathematical models, which reduces the scientific nature of the measurement results.	This research constructs a comprehensive indicator framework for evaluating the emergency logistics responsiveness of various regions in China. The framework integrates multiple key factors affecting emergency logistics and uses the entropy weight TOPSIS method for evaluation.
Differences in regional logistics industry development	Existing research mainly focuses on regional logistics development, but the development of the logistics industry may not necessarily improve emergency responsiveness. Due to the differences in operation, organization and participation between social logistics and emergency logistics, there are differences in emergency logistics responsiveness in different regions, and relevant research rarely explores the causes.	This research uses an empirical approach to identify the causes of regional differences in emergency logistics responsiveness in China.

## 3 Measurement of China’s regional emergency logistics responsiveness and analysis of regional differences

### 3.1 Constituent factors and measurement indexes of emergency logistics responsiveness

Emergency logistics, like general logistics, consists of factors such as fluid carrier, flow direction, and flow rate (23). In the empirical research on logistics and emergency logistics capacity, Experts have reached some certain consensus on the measurement indexes of logistics flow rate capacity, logistics carrier carrying capacity, and logistics flow capacity (16, 24, 25). Therefore, this study refers to the definition, element composition, and existing research ideas and methods of emergency logistics. Based on Xi’s (26) analysis of the selection of elements and indexes of emergency logistics capacity, the composition index system of emergency logistics responsiveness is summarized into emergency logistics flow responsiveness, emergency logistics carrier carrying capacity, emergency logistics flow effectiveness responsiveness, emergency logistics flow rate responsiveness, regional information responsiveness, and other indexes. Since governments manage and dispatch emergency logistics under public emergencies at all levels, this paper introduces relevant indexes of government early warning guarantee capacity as the components of emergency logistics responsiveness (27). The specific index system is shown in Table 3.

**Table 3:** Components of emergency logistics responsiveness factors and index system.

First level index	Second level index	Third level index	Index property
Emergency logistics flow responsiveness	Freight turnover	Railroad freight turnover	+
		Road freight turnover	+
	Freight volume	Road freight	+
		Railroad freight	+
Emergency logistics carrier carrying capacity	Practitioners in the logistics industry	Aviation, road, rail, stevedoring, and other postal, warehousing, and transport employment	+
	Logistics and transport equipment ownership	Civil vehicle ownership	+
		Road operation vehicle ownership	+
	Logistics infrastructure	Transport network density	+
	Logistics carrier capacity	Express delivery volume	+
Emergency logistics flow effectiveness responsiveness	Unit capital investment completed commodity turnover rate	Freight volume and logistics industry capital investment ratio	+
		Freight turnover and logistics industry capital investment ratio	+
	Material logistics volume per unit of manpower	Freight turnover to employment ratio	+
		Freight volume to employment ratio	+
Emergency logistics flow rate responsiveness	Share of graded roads	Percentage of primary roads	+
		Percentage of secondary roads	+
		Percentage of highways	+
Regional informatization responsiveness	Regional communication level	Number of ports for Internet broadband access	+
		Number of mobile phone users	+
	Network infrastructure	Fiber optic line length	+
	Telecommunications business volume	Volume of postal and telecommunication operations	+
Social emergency guarantee capacity	Regional policy support	Share of local transportation expenditures in total local fiscal expenditures	+
	Rescue guarantee foundation	Number of beds in health facilities	+
		Number of CDCs	+
		Number of health technical personnel	+

The samples observed in this study include 30 provinces (municipalities and autonomous regions) in China. Due to the difficulty in obtaining relevant data, the research samples do not include Hong Kong, Taiwan, Macao, and Tibet. The sample observation period is from 2012 to 2021. All original data are from the China Statistical Yearbook^1^ of the corresponding year and the website of the National Bureau of Statistics.^2^ Some indexes are calculated from raw data, such as the proportion of fixed asset investment in the logistics industry in social fixed asset investment. For individual missing data, interpolation or analogy replaces the missing values.

### 3.2 Emergency logistics responsiveness measurement model

Previous studies on logistics capacities have mainly used the analytical hierarchy process (AHP) (28), fuzzy comprehensive evaluation (FCE) (29), gray relational analysis (GRA) (30), and multi-criteria optimization and compromise solution (VIKOR) (31). Table 4 details the advantages and disadvantages of the above MDCM tools and the reasons why they are not suitable for this paper.

**Table 4:** Comparison of MDCM tools.

Methods	Overview	Advantages	Disadvantages	Reasons for not being suitable for this paper
Analytical Hierarchy Process (AHP)	Determines priorities by pairwise comparison of hierarchical indicators.	Simple structure, easy to implement, suitable for complex decision-making problems; highly flexible.	Weighting relies on subjective judgment, which may lead to deviations	This paper requires objective quantitative indicator weights, but AHP relies on subjective weighting, which is not scientific enough.
Fuzzy Comprehensive Evaluation (FCE)	Based on fuzzy mathematics theory, fuzzy matrix is used to deal with uncertainty problems.	It is suitable for dealing with highly ambiguous problems and can combine qualitative and quantitative indicators.	Weight allocation still requires subjective judgment; the calculation is complex and the results are difficult to interpret.	It tends to be qualitative analysis and cannot meet the needs of this paper for quantitative and objective evaluation.
Gray Relational Analysis (GRA)	Compare the trend of sequence changes and analyze the correlation of various indicators.	Suitable for problems with incomplete data or small samples; can reveal the correlation between indicators.	It tends to analyze correlations and has difficulty providing comprehensive rankings; it is sensitive to data fluctuations.	This paper needs to rank the regional emergency logistics responsiveness comprehensively. GRA can only analyze the correlation.
Multi-Criteria Optimization and Compromise Solution (VIKOR)	Evaluates solutions by balancing ideal and satisfactory outcomes through compromise.	Emphasizes compromise and is suitable for scenarios that require trade-offs; the calculation is relatively simple.	Biased conflict indicator analysis, sensitive to weights; complex calculations.	It mainly solves the problem of indicator conflicts, which is inconsistent with the comprehensive evaluation goal of this paper.

Therefore, this study uses the entropy weight TOPSIS method to measure China’s emergency logistics responsiveness from 2012 to 2021. Firstly, the entropy weight method objectively assigns weights based on the degree of discreteness of the data, which can effectively avoid the human bias that may be introduced by the subjective weighting method. This improves the scientificity and objectivity of the evaluation results, thereby more accurately reflecting the fundamental differences between regions. Secondly, the TOPSIS method can comprehensively evaluate the quality of regional emergency logistics responsiveness based on the distance calculation from the ideal solution and the negative ideal solution, meeting the needs of quantitative comparison and ranking of various regions. In addition, this method is highly adaptable in data processing and solves the comparability problem of data in different dimensions through indicator standardization. This enables the constructed evaluation index system to reflect the key characteristics of emergency logistics responsiveness more systematically. Finally, the combination of the entropy weight method and TOPSIS method not only ensures the objectivity of weight distribution but also can intuitively measure the performance of each region through the proximity to the ideal solution. Therefore, the entropy weight TOPSIS method meets the dual needs of objectivity and comprehensiveness in research. The entropy weight TOPSIS method determines the weight based on the entropy weight method. Then, it uses the Euclidean distance to obtain the relative closeness of each evaluation object to the positive ideal solution. This closeness is used as the basis for evaluation and ranking. The specific calculation steps are as follows:

1. Standardization of original data. The data are dimensionless to facilitate the comparison of indexes of different provinces in different years. Finally, the measurement value of the emergency logistics responsiveness of every province in China is obtained. The indexes selected in this paper are all positive values. The data processing is shown in Formula 3-1:

(3-1) $$x_{\theta ij}^{'}\;=\frac{x_{\theta ij}-\min\left\{x_{\theta 1j},\ldots ,\;x_{\theta nj}\right\}}{\max\left\{x_{\theta 1j},\ldots ,\;x_{\theta nj}\right\}-\min\left\{x_{\theta 1j},\ldots ,\;x_{\theta nj}\right\}}$$

Where: assuming *r* years, *n* provinces (cities, districts), *m* indexes, then $x_{\theta ij}$ stands for the value of the *j-th* index of province (city, district) *i* in year *θ*, of which θ=1,2,…,r;i=1,2,…,n;j=1,2…,m . $x_{\theta ij}$ is the initialized value of the index; $x_{\theta ij}^{\prime }$ is the standardized value of the index.

2. Calculation of weights
Calculate the characteristic specific gravity value, as shown in Formula 3-2:

(3-2) $$p_{\theta ij}=\frac{x_{\theta ij}^{'}}{\sum_{\theta =1}^{r}\sum_{i=1}^{n}x_{\theta ij}^{'}}$$

Among them, $p_{\theta ij}$ represents the characteristic weight value of the *j-th* index in the *i* province (region, city).

2. Solve the information entropy $e_{j}$ of the *j-th* index, as shown in Formula 3-3:

(3-3) $$e_{j}=\frac{\sum_{\theta =1}^{r}\sum_{i=1}^{n}\left(p_{\theta ij}*\ln p_{\theta ij}\right)}{\ln rn}$$

3. Calculate the weight $w_{j}$ of the j-th index, as shown in Formula 3-4:

(3-4) $$w_{j}=\frac{1-e_{j}}{\sum_{j=1}^{n}\left(1-e_{j}\right)}$$

3. Create the weight matrix Z

$Z={\left(z_{\theta ij}\right)}_{r\times m\times n},\;z_{\theta ij}=w_{j}\times x_{\theta ij}^{\prime }\left(\theta =1,2,\ldots ,r;i=1,2,\ldots ,n;j=1,2,\ldots ,m\right)$

*Z* is the weighted decision matrix consisting of all the weighted decision scores; $z_{\theta ij}$ denotes the weighted decision score.

4. Calculate the Euclidean distance between the actual level of emergency logistics responsiveness of each province and the positive and negative ideal solutions, as shown in Formulas 3-5, 3-6:

(3-5) $$d_{j}^{+}=\max\left(z_{\theta ij}\right),\;D_{\theta_{i}}^{+}=\sqrt{\overset{n}{\underset{j=1}{\sum }}{\left(d_{j}^{+}-z_{\theta ij}\right)}^{2}}$$

(3-6) $$d_{j}^{-}=\min\left(z_{\theta ij}\right),\;D_{\theta_{i}}^{-}=\sqrt{\overset{n}{\underset{j=1}{\sum }}{\left(d_{j}^{-}-z_{\theta ij}\right)}^{2}}$$

Among them, $d_{j}^{+}$ and $d_{j}^{-}$ are the positive ideal solution and the negative ideal solution, respectively. The positive ideal solution represents the region with the most ideal development of emergency logistics responsiveness in each province and city; the negative ideal solution represents the region with the least ideal development of emergency logistics responsiveness in each province and city. $D_{\theta i}^{+}$ and $D_{\theta i}^{-}$ represent the Euclidean distance between the actual level of emergency logistics responsiveness of each province and the positive and negative ideal solutions, respectively.

5. Calculate the emergency logistics responsiveness of each province, as shown in Formula 3-7:

(3-7) $$C_{\theta i}=\frac{D_{\theta i}^{-}}{D_{\theta i}^{+}+D_{\theta i}^{-}}$$

Among them, $C_{\theta i}\in \left[0,1\right]$ , the closer the $C_{\theta i}$ value is to 1, the higher the emergency logistics responsiveness of the region; conversely, the closer the $C_{\theta i}$ value is to 0, the lower the emergency logistics responsiveness of the region.

### 3.3 Dagum Gini coefficient

Compared with the traditional Gini coefficient, coefficient of variation, and Theil index, the Dagum Gini coefficient can fully consider the distribution of sub-samples and the problems of cross-overlap between samples (32). At the same time, the Dagum Gini coefficient can effectively solve the issue of the source of regional differences. Therefore, it has excellent advantages in analyzing spatial imbalance (33). The Dagum Gini coefficient is used to characterize the problem of regional development imbalance. It is widely used in research fields such as regional disparity in residents’ income and regional disparity in economic development (34, 35). It is also an essential tool for this paper to study the regional differences in China’s emergency logistics responsiveness. The calculation formula of the Dagum Gini coefficient is shown in Formula 3-8:


(3-8) $$G=\frac{\overset{k}{\underset{j=1}{\sum }}\overset{k}{\underset{h=1}{\sum }}\overset{k}{\underset{i=1}{\sum }}\overset{k}{\underset{r=1}{\sum }}|y_{ji}-y_{\mathrm{hr}}|}{2\mu n^{2}}$$

Among them, *G* is the overall Gini coefficient, *n* is the number of provinces (regions, cities), *k* is the number of regions, *i* and *r* represent the number of provinces (districts, cities) in the region. $n_{j}\left(n_{h}\right)$ represents the number of provinces (regions, cities) in region *j*(*h*). $y_{ji}\left(y_{\mathrm{hr}}\right)$ represents the emergency logistics responsiveness value of the *i*(*r*)-th province (region, city) in the *j*(*h*) region. μ represents the means of emergency logistics responsiveness of each province.

In addition, the Dagum Gini coefficient decomposition method by subgroup can further decompose the overall Gini coefficient (*G*) into the following three parts: intra-regional variability ( $G_{w}$ ), inter-regional variability ( $G_{nb}$ ), and hypervariability density ( $G_{t}$ ). The calculation formulas are shown in Formulas 3-9, 3-10:


(3-9) $$\begin{array}{l} G=\overset{k}{\underset{j=1}{\sum }}G_{jj}p_{j}s_{j}+\overset{k}{\underset{j=2}{\sum }}\overset{j-1}{\underset{h=1}{\sum }}G_{jh}\left(p_{j}s_{h}+p_{h}s_{j}\right)D_{jh}\\ +\overset{k}{\underset{j=2}{\sum }}\overset{j-1}{\underset{h=1}{\sum }}G_{jh}\left(p_{j}s_{h}+p_{h}s_{j}\right)\left(1-D_{jh}\right) \end{array}$$

(3-10) $$G=G_{w}+G_{nb}+G$$

Among them, $G_{w}$ represents the difference in emergency logistics responsiveness within region *j*(*h*). $G_{nb}$ represents the difference in emergency logistics responsiveness between regions *j*(*h*). $G_{t}$ represents the residual term of cross-overlapping emergency logistics responsiveness between regions. $G_{jj}$ represents the Gini coefficient within region *j*, $G_{jh}$ represents the inter-regional Gini coefficient between region *j* and region *h*, and $D_{jh}$ represents the relative influence of the emergency logistics responsiveness between regions *j* and *h*. Formulas 3-11–3-15 show the corresponding calculation formulas. $p_{j}=n_{j}/n$ represents the ratio of the number of provinces $n_{j}$ in region *j* to the sample size *n*, and $s_{j}=n_{j}\mu_{j}/n\mu$ represents the ratio of the emergency logistics responsiveness in region *j* to all provinces’ emergency logistics responsiveness.


(3-11) $$G_{jj}=\frac{\frac{1}{2{\bar{Y}}_{j}}\overset{n_{j}}{\underset{i=1}{\sum }}\overset{n_{j}}{\underset{r=1}{\sum }}|y_{ji}-y_{jr}|}{n_{j}^{2}}$$

(3-12) $$G_{jj}=\frac{\frac{1}{2{\bar{Y}}_{j}}\overset{n_{j}}{\underset{i=1}{\sum }}\overset{n_{j}}{\underset{r=1}{\sum }}|y_{ji}-y_{jr}|}{n_{j}^{2}}$$

(3-13) $$D_{jh}=\frac{d_{jh}-p_{jh}}{d_{jh}+p_{jh}}$$

(3-14) $$d_{jh}=\overset{\infty }{\underset{0}{\int }}dF_{j}\left(y\right)\overset{y}{\underset{0}{\int }}\left(y-x\right)dF_{h}\left(x\right)$$

(3-15) $$p_{jh}=\overset{\infty }{\underset{0}{\int }}dF_{h}\left(y\right)\overset{y}{\underset{0}{\int }}\left(y-x\right)dF_{j}\left(x\right)$$

Among them, $\mu_{j}$ and $\mu_{h}$ represent the mean values of emergency logistics responsiveness in regions *j* and *h*. $d_{jh}$ represents the difference between the emergency logistics responsiveness of regions *j* and *h*. $p_{jh}$ represents the hypervariable matrix between regions *j* and *h*, and $F_{h}\left(F_{j}\right)$ represents the cumulative distribution function of the emergency logistics responsiveness of region (*h*) *j*.

### 3.4 Results of the measurement of China’s regional emergency logistics responsiveness

Based on the above index system, this study uses Stata 17 software. It uses the entropy weight TOPSIS method to measure the emergency logistics responsiveness of 30 provinces (autonomous regions and municipalities) in China from 2012 to 2021. The entropy method is used to calculate the weights of each measurement index, as shown in Table 5. The measurement results are shown in Table 6. Firstly, by comparing the measurement values of each province from 2012 to 2021, it can be seen that the emergency logistics responsiveness of each province in China has been significantly improved. Secondly, it can be seen from the provincial average that Qinghai Province had the lowest emergency logistics responsiveness during the sample observation period, with a mean value of only 0.0552. Guangdong Province has the most robust emergency logistics responsiveness, with a mean value of 0.4696. It shows apparent gaps in the emergency logistics responsiveness of various provinces in China.

**Table 5:** Summary of entropy weight calculation results.

Indexes	Weight coefficient *w*	Information utility value *d*	Information entropy value *e*
Railroad freight turnover	8.16%	0.1069	0.8931
Road freight turnover	5.02%	0.0657	0.9343
Road freight	3.03%	0.0397	0.9603
Railroad freight	8.29%	0.1085	0.8915
Aviation, road, rail, stevedoring, and other postal, warehousing, and transport employment	2.33%	0.0305	0.9695
Civil vehicle ownership	3.69%	0.0483	0.9517
Road operation vehicle ownership	2.79%	0.0366	0.9634
Transport network density	3.60%	0.0472	0.9528
Express delivery volume	15.90%	0.2082	0.7918
Freight volume and logistics industry capital investment ratio	3.18%	0.0417	0.9583
Freight turnover and logistics industry capital investment ratio	6.80%	0.089	0.911
Freight turnover to employment ratio	1.91%	0.025	0.975
Freight volume to employment ratio	2.59%	0.0339	0.9661
Percentage of primary roads	4.27%	0.0559	0.9441
Percentage of secondary roads	1.28%	0.0167	0.9833
Percentage of highways	0.77%	0.0101	0.9899
Number of ports for Internet broadband access	3.78%	0.0495	0.9505
Number of mobile phone users	3.01%	0.0395	0.9605
Fiber optic line length	3.62%	0.0475	0.9525
Volume of postal and telecommunication operations	8.20%	0.1074	0.8926
Share of local transportation expenditures in total local fiscal expenditures	0.78%	0.0103	0.9897
Number of beds in health facilities	2.60%	0.0341	0.9659
Number of CDCs	1.90%	0.0249	0.9751
Number of health technical personnel	2.51%	0.0328	0.9672

**Table 6:** Calculation results of emergency logistics responsiveness in various provinces in China.

Province	2012	2013	2014	2015	2016	2017	2018	2019	2020	2021	Average
Jiangsu	0.2158	0.2487	0.2612	0.2730	0.2840	0.3066	0.3320	0.3686	0.3960	0.4274	0.3113
Hubei	0.1306	0.1482	0.1566	0.1654	0.1709	0.1803	0.1939	0.1926	0.1929	0.2027	0.1734
Hunan	0.1472	0.1497	0.1576	0.1591	0.1650	0.1783	0.1901	0.1879	0.2010	0.2091	0.1745
Xinjiang	0.1152	0.1192	0.1199	0.1174	0.1186	0.1228	0.1408	0.1379	0.1359	0.1389	0.1267
Shandong	0.2811	0.2795	0.2837	0.2898	0.3110	0.3250	0.3509	0.3686	0.3958	0.4133	0.3299
Ningxia	0.0981	0.0836	0.0794	0.0791	0.0798	0.0808	0.0833	0.0925	0.0991	0.0995	0.0875
Gansu	0.0892	0.0931	0.0931	0.0927	0.0944	0.1014	0.1120	0.1113	0.1127	0.1161	0.1016
Heilongjiang	0.1348	0.1383	0.1363	0.1321	0.1359	0.1441	0.1430	0.1481	0.1465	0.1481	0.1407
Shanghai	0.2206	0.2406	0.2663	0.2540	0.2570	0.2807	0.2916	0.3220	0.3406	0.3600	0.2833
Beijing	0.1938	0.2029	0.2033	0.2029	0.2126	0.2186	0.2252	0.2280	0.2451	0.2525	0.2185
Zhejiang	0.1893	0.1991	0.2145	0.2348	0.2498	0.2740	0.3011	0.3354	0.3800	0.4147	0.2793
Yunnan	0.0993	0.1223	0.1234	0.1270	0.1305	0.1402	0.1476	0.1530	0.1612	0.1716	0.1376
Shaanxi	0.1413	0.1531	0.1656	0.1621	0.1700	0.1801	0.1901	0.1934	0.2056	0.2156	0.1777
Jiangxi	0.1269	0.1359	0.1361	0.1326	0.1362	0.1561	0.1670	0.1567	0.1655	0.1714	0.1484
Qinghai	0.0490	0.0482	0.0513	0.0548	0.0533	0.0520	0.0587	0.0591	0.0619	0.0638	0.0552
Sichuan	0.1555	0.1866	0.1964	0.2035	0.2119	0.2229	0.2486	0.2513	0.2672	0.2863	0.2230
Tianjin	0.1425	0.1299	0.1333	0.1282	0.1301	0.1340	0.1368	0.1418	0.1433	0.1435	0.1363
Hebei	0.2388	0.2533	0.2656	0.2637	0.2758	0.2981	0.3211	0.3317	0.3542	0.3722	0.2975
Guangdong	0.2887	0.3699	0.3875	0.4058	0.4310	0.4755	0.5357	0.5531	0.5954	0.6532	0.4696
Guangxi	0.1346	0.1397	0.1438	0.1445	0.1490	0.1609	0.1741	0.1764	0.1932	0.2022	0.1618
Liaoning	0.2176	0.2210	0.2249	0.2258	0.2489	0.2614	0.2815	0.2705	0.2579	0.2637	0.2473
Chongqing	0.0799	0.0897	0.0926	0.0960	0.0989	0.1067	0.1123	0.1141	0.1181	0.1241	0.1032
Henan	0.2895	0.2725	0.2858	0.2879	0.3017	0.3174	0.3380	0.3433	0.3634	0.3741	0.3174
Guizhou	0.0759	0.0849	0.0915	0.0944	0.0978	0.1045	0.1160	0.1168	0.1228	0.1305	0.1035
Anhui	0.2178	0.2223	0.2271	0.1980	0.2025	0.2169	0.2354	0.2270	0.2380	0.2412	0.2226
Fujian	0.1194	0.1358	0.1403	0.1470	0.1497	0.1643	0.1806	0.1892	0.1971	0.2100	0.1633
Shanxi	0.1793	0.1882	0.1967	0.1922	0.1941	0.2322	0.2462	0.2518	0.2579	0.2703	0.2209
Jilin	0.1017	0.0982	0.0985	0.0951	0.0979	0.1051	0.1101	0.1128	0.1197	0.1222	0.1061
Hainan	0.0683	0.0444	0.0538	0.0531	0.0516	0.0508	0.0536	0.0687	0.0855	0.0896	0.0619
Inner Mongolia	0.1908	0.1835	0.1878	0.1791	0.1834	0.2108	0.2330	0.2216	0.2180	0.2222	0.2030

**Figure 1:**
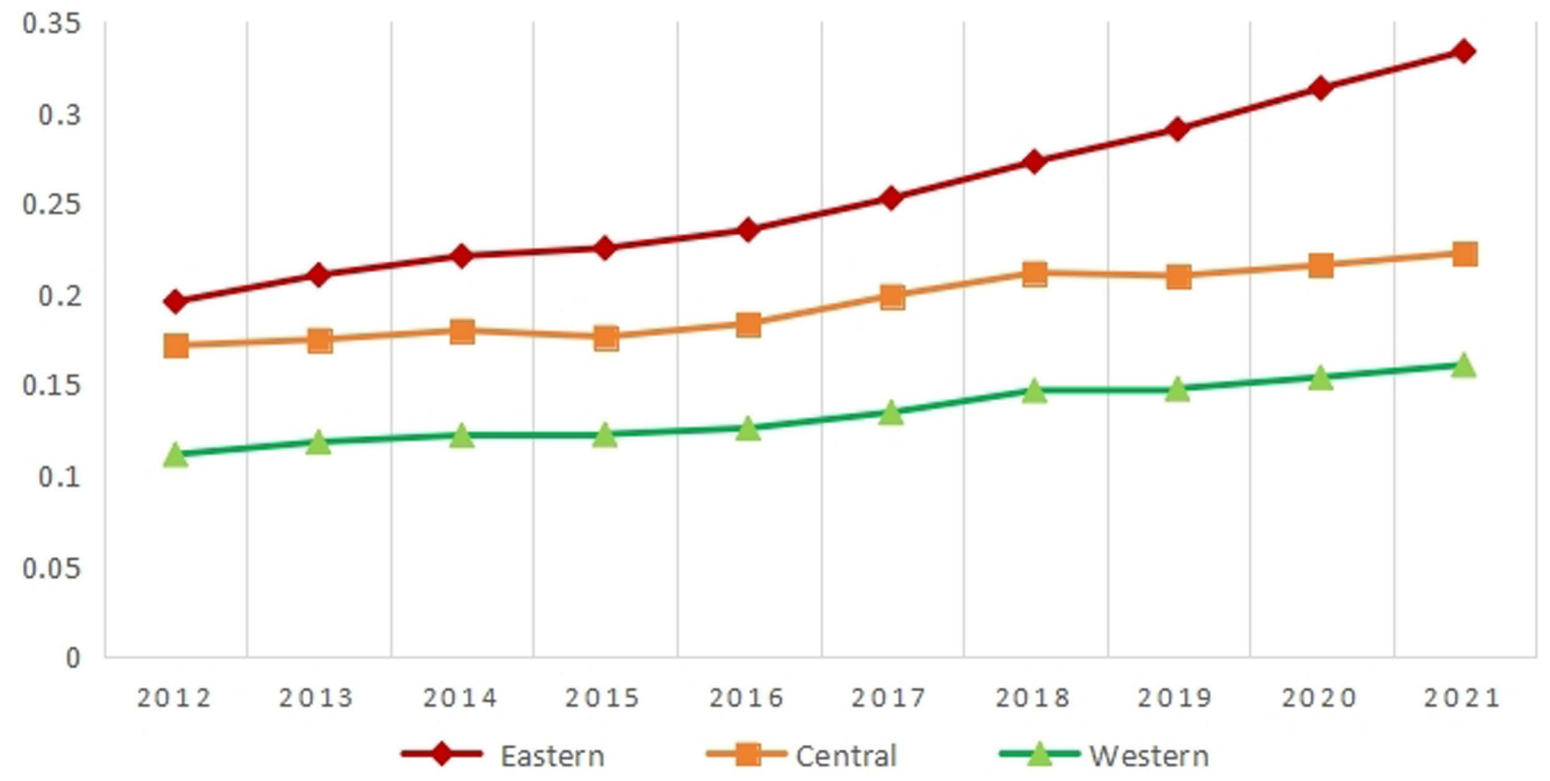
Dynamic changes in China’s emergency logistics responsiveness from 2012 to 2021.

To more comprehensively grasp regional emergency logistics development trends, refer to the standards released by the National Bureau of Statistics of China in 2003 (36). The standard considers economic development and geographical factors and divides the 30 provinces (autonomous regions, municipalities) into three major economic regions: western, central, and eastern.^3^ The results of the measurement and analysis of the emergency logistics responsiveness of the three regions and the country are shown in Table 7 and Figure 1. From a national level, China has significantly grown emergency logistics responsiveness from 2012 to 2021. From 0.1577 in 2012 to 0.2370 in 2021, the overall growth rate reached 50.29%. This growth was particularly significant between 2017 and 2018. This growth may be directly related to the establishment of the Emergency Management Department in 2018. Ministry of Emergency Management has also continuously released corresponding plans to construct and comprehensively deploy emergency logistics as a critical project. As a result, China’s emergency logistics responsiveness has been rapidly improved. From a regional perspective, the emergency logistics responsiveness in western, central, and eastern regions has increased steadily. The emergency logistics responsiveness in eastern China is the strongest, followed by central China, and western China is the weakest. The central and eastern regions’ emergency logistics responsiveness is higher than the national average. The emergency logistics responsiveness in the western region is relatively low, below the national average.

**Table 7:** Results of the measurement of emergency logistics responsiveness in various regions of China.

Years	Western	Central	Eastern
2012	0.11171	0.17171	0.19583
2013	0.11853	0.17492	0.21041
2014	0.12226	0.17996	0.22095
2015	0.12278	0.17647	0.22523
2016	0.12614	0.18368	0.23526
2017	0.13482	0.19909	0.25276
2018	0.14696	0.21169	0.27286
2019	0.14794	0.21009	0.29071
2020	0.15416	0.21587	0.3133
2021	0.16098	0.22253	0.33364

### 3.5 Regional differences and decomposition of China’s emergency logistics responsiveness

This paper uses the Dagum Gini coefficient and its subgroup decomposition method to further decompose the overall variability into intra-regional variability, inter-regional variability, and inter-regional hypervariability density. The specific measurement results are shown in Table 8.

**Table 8:** Regional differences in China’s emergency logistics responsiveness and their decomposition results.

Years		2012	2013	2014	2015	2016	2017	2018	2019	2020	2021
Overall Gini coefficient		0.234	0.247	0.251	0.254	0.261	0.265	0.269	0.274	0.279	0.287
Decomposition item	Intra-regional differences	0.054	0.058	0.059	0.059	0.061	0.062	0.064	0.064	0.065	0.067
	Inter-regional differences	0.126	0.130	0.135	0.139	0.141	0.143	0.142	0.155	0.164	0.170
	Ultra variable density	0.054	0.058	0.056	0.055	0.058	0.059	0.062	0.054	0.049	0.050

During the observation period, the overall Gini coefficient of China’s emergency logistics responsiveness increased yearly from 0.2347 in 2012 to 0.2875 in 2021. This shows that the overall diversity of China’s emergency logistics responsiveness is expanding. In terms of decomposition items, except for a few years, the intra-regional diversity, inter-regional diversity, and super-variable density have continuously increased. The values of intra-regional diversity and super-variable density are small. The inter-regional diversity is significantly greater. However, all regions in China have issued emergency plans and relevant policies on emergency logistics. However, each region’s economic development level, logistics system infrastructure, and other conditions differ significantly. The differences in emergency logistics responsiveness between regions have increased accordingly, which has led to a further increase in overall differences.

Combined with the above analysis of the measurement and differences in China’s regional emergency logistics responsiveness, it is found that the differences in emergency logistics responsiveness between regions in China are in a trend of continuous expansion. So, what are the regional differences in China’s potential emergency logistics responsiveness under the impact of public emergencies? In response to this issue, the following content conducts an empirical study.

### 3.6 Regional differences in emergency logistics responsiveness under the impact of public emergencies

#### 3.6.1 Variable selection

To achieve the purpose of the study, this study defines emergency logistics responsiveness (*logistic*) as the dependent variable and uses data from 2012 to 2021 for analysis. The data used are obtained based on the entropy weight TOPSIS method described in Section 3.3.

To reveal the differences in emergency logistics responsiveness in different regions under the impact of public emergencies, this study chose the scale of public emergencies (*accident*) as the independent variable. This variable measures the cumulative number of geological disasters, fires, and environmental crises. The data is collected from the yearbook published by the National Bureau of Statistics of China (see text footnote 1), and the period covered is also from 2012 to 2021. Please refer to Table 9 for the specific definition and detailed description of the variables.

**Table 9:** Variable definition brief description.

Variable type	Variable symbol	Variable name	Index measurement
Dependent variable	*logistic*	Emergency logistics responsiveness	The entropy weight TOPSIS method was used to measure the results.
Independent variable	*accident*	The scale of public emergencies	The total number of environmental crises, geological disasters, and fires was used as a proxy variable.

#### 3.6.2 Model construction

According to the research objectives, this study established *logistic* as the dependent variable and included *accident* as the independent variable in the model. The regional differences in emergency logistics responsiveness under the impact of public emergencies are explored by establishing a panel quantile regression model. The expression of Formula 3-16 is as follows:


(3-16) $$\mathrm{lnlogistic}=\alpha_{1q}+b_{2q}\;\mathrm{lnacciden}t_{tq}+\delta_{\mathrm{it}}$$

In models (Formula 3-16), lnlogistic stands for the emergency logistics responsiveness; $\mathrm{lnacciden}t_{tq}$ stands for the scale of public emergencies at the three quantiles of 0.25, 0.5, and 0.75; the random disturbance term $\delta_{\mathrm{it}}$ is used to capture the impact of unobserved factors; *it* stands for the sample of a province (municipality, autonomous region) in China at a particular time from 2012 to 2021.

#### 3.6.3 Impact of public emergencies on emergency logistics responsiveness

**Figure 2:**
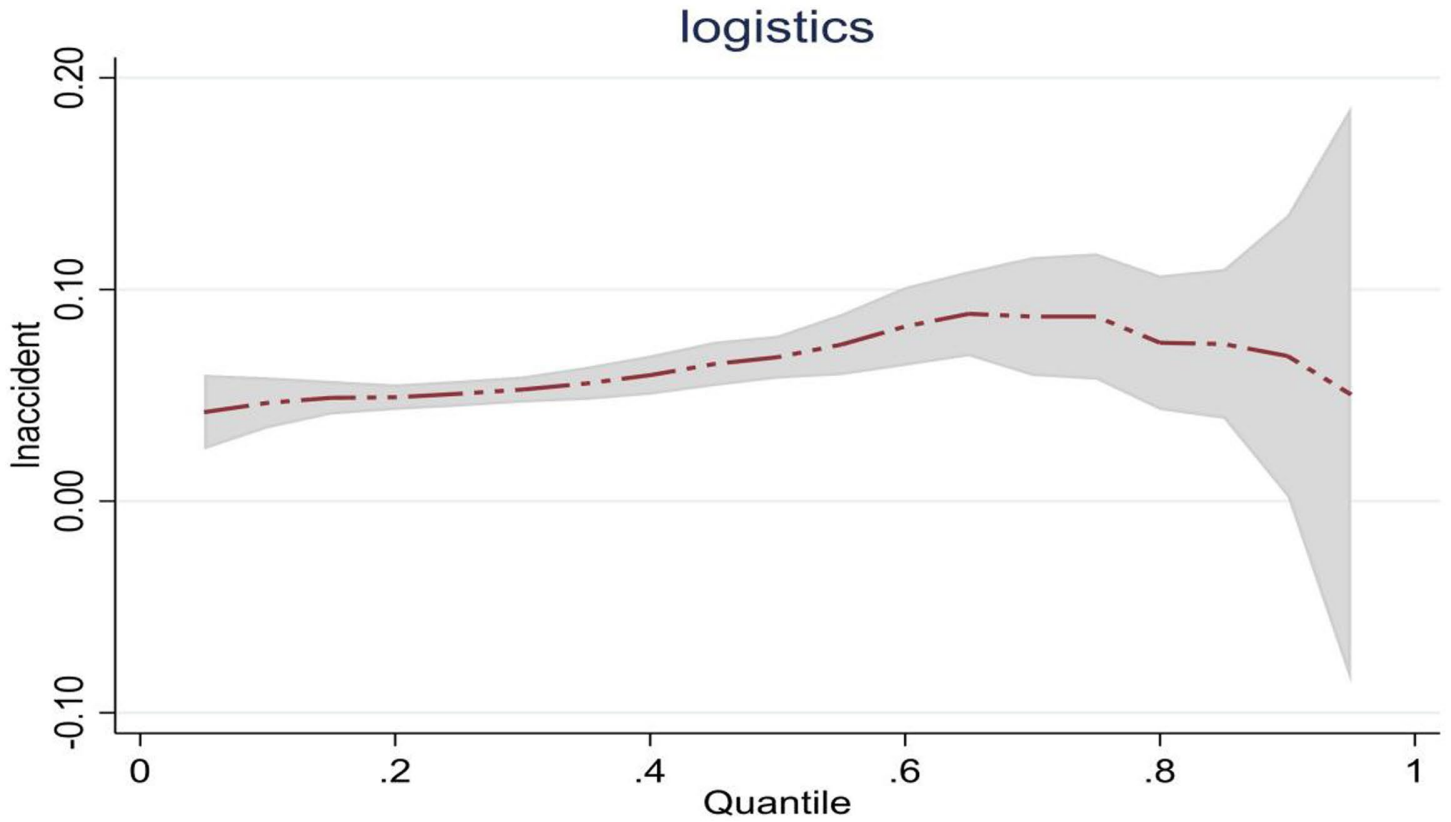
Dynamic changes of emergency logistics responsiveness in China under the impact of public emergencies.

Considering the panel data analyzed in this paper is a short panel, there is no need to conduct stationarity and cointegration tests during regression. The model’s semi-logarithmic panel quantile regression analysis method was used to conduct regression analysis on the national and three primary regional samples. The results of linear regression are shown in Table 10. Observation from national samples, the estimated coefficients of *lnaccident* at quantiles 0.25, 0.5, and 0.75 are all positive and significant, and the effect change on *lnlogistics* continues to increase from 0.051 to 0.087. This shows that China’s emergency logistics responsiveness will be promoted as the scale of public emergencies expands. On the other hand, it implies the effectiveness of emergency management agencies in converting general logistics resources and optimizing emergency plans. However, judging from the influence trend, the influence of expanding the scale of public emergencies on emergency logistics responsiveness shows a trend of first increasing and then gradually decreasing. This changing trend is shown in Figure 2. It reflects that when the scale expansion of public emergencies is high, the complexity of public emergencies may continue to increase. It may be affected by limited logistics resources, and the emergency management agency cannot convert general logistics into emergency logistics, leading to emergency logistics responsiveness gradually declining.

**Table 10:** Regional differences in China’s emergency logistics responsiveness under the impact of public emergencies.

Brochure	Variable	QR_0.25	QR_0.5	QR_0.75
National	*lnaccident*	0.051*** (0.0038)	0.068*** (0.0080)	0.087*** (0.012)
	Intercept (C)	−0.306*** (0.032)	−0.422*** (0.0700)	−0.522*** (0.103)
Western	*lnaccident*	0.040*** (0.0076)	0.053*** (0.0096)	0.078*** (0.0130)
	Intercept (C)	−0.225*** (0.063)	−0.312*** (0.0801)	−0.468*** (0.109)
Central	*lnaccident*	0.030*** (0.0098)	0.017 (0.045)	0.046** (0.022)
	Intercept (C)	−0.114*** (0.087)	0.045 (0.146)	−0.168 (0.197)
Eastern	*lnaccident*	0.089*** (0.017)	0.050** (0.022)	0.0399** (0.016)
	Intercept (C)	−0.626 (−0.626)	−0.186 (0.193)	−0.019 (0.142)

*** indicates significant at the 1% level; ** indicates significant at the 5% level;* indicates significant at the 10% level.

**Figure 3:**
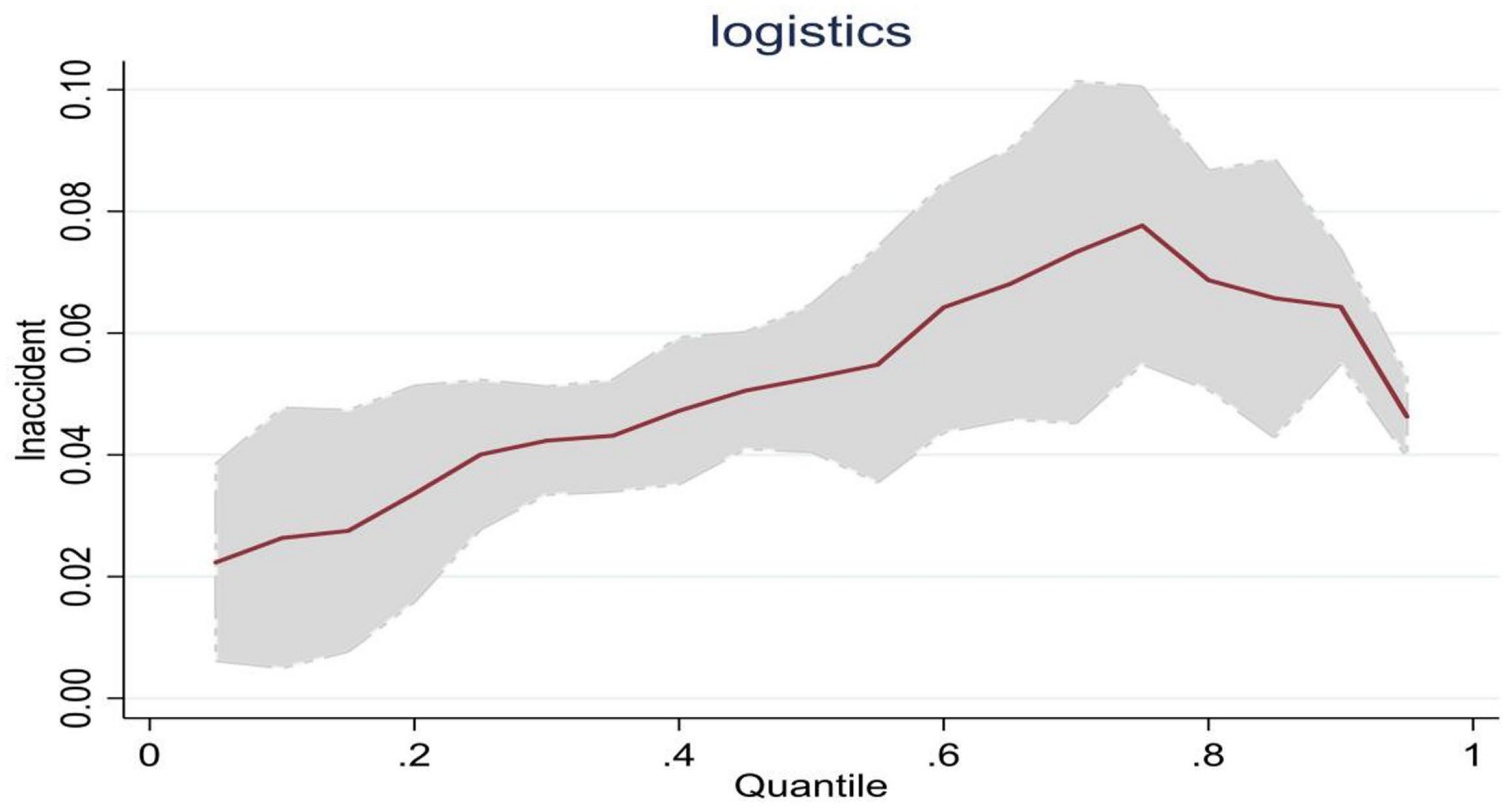
Dynamic changes in emergency logistics responsiveness in Western China under the impact of public emergencies.

When analyzing the *lnaccident* estimated coefficients in western, central, and eastern China, it was found that they were all significant and positive at the 0.25, 0.5, and 0.75 quantiles, but the change trends in each region were different. Especially in western China, its coefficient rose from 0.040 to 0.078. This changing trend is shown in Figure 3, reflecting that as the scale of public emergencies grows, the region’s emergency logistics responsiveness steadily improves. This reflects that the emergency management agencies in western China can effectively convert general logistics into emergency logistics in responding to public emergencies. In addition, they can continuously summarize previous experience and improve emergency management capacities in response to public emergencies, thereby continuously enhancing emergency logistics responsiveness.

**Figure 4:**
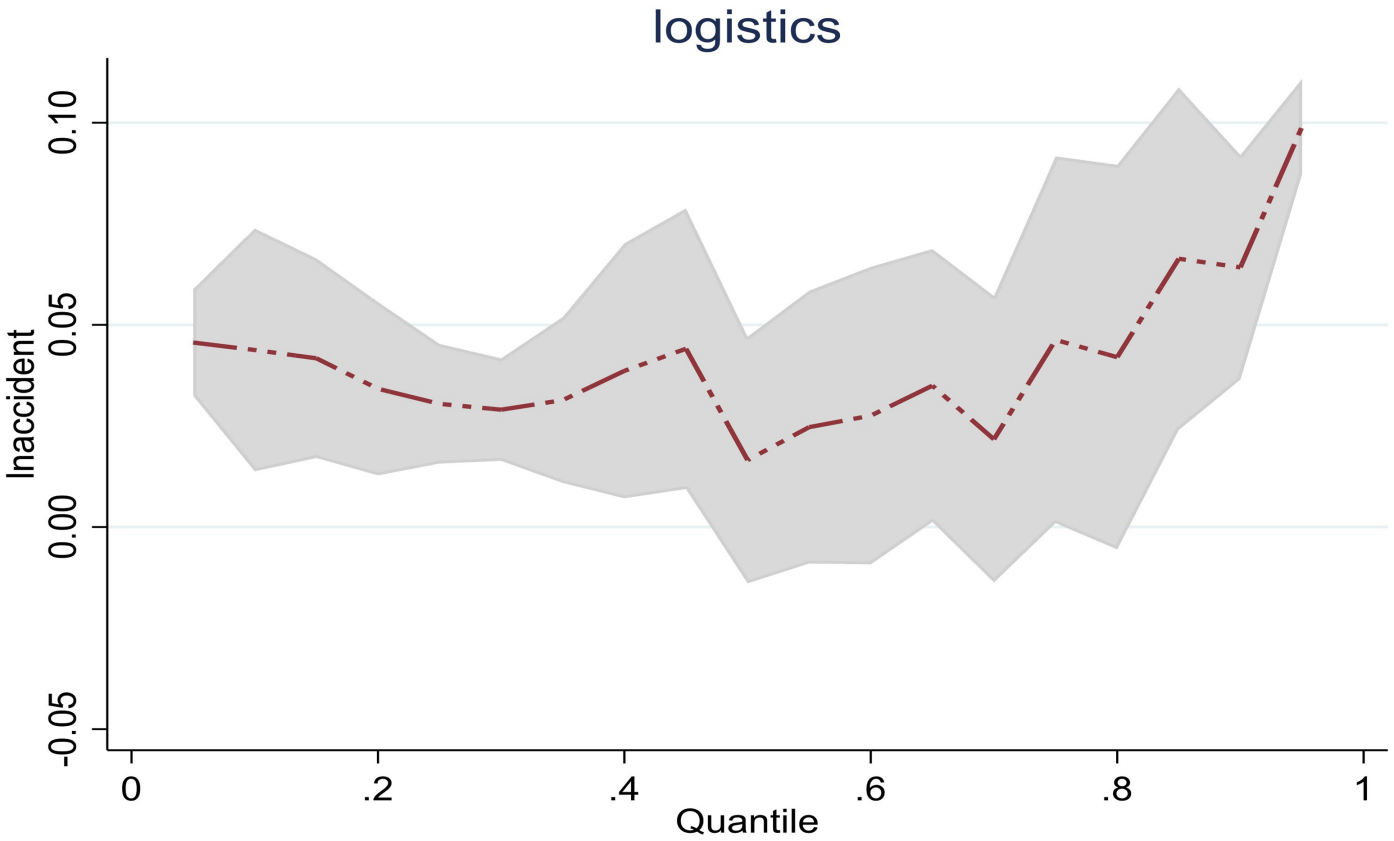
Dynamic changes of emergency logistics responsiveness in Central China under the impact of public emergencies.

The estimated coefficients of the central region at the 0.25, 0.5, and 0.75 quantiles are 0.031, 0.017, and 0.046, respectively. It can be seen that as the scale of public emergencies expands, the dynamic changes in the emergency logistics responsiveness in central China show a trend of decreasing first and then increasing. This changing trend is shown in Figure 4. This reflects that in terms of responding to the impact of public emergencies, as the scale of public emergencies continues to expand, the efficiency of the emergency management agencies in central China in converting social logistics into emergency logistics may be low. This has resulted in a decline in emergency logistics responsiveness. However, in the continuous response to public emergencies, central China may have a strong learning ability, improve the efficiency of converting general logistics into emergency logistics, and thus improve the emergency logistics responsiveness.

**Figure 5:**
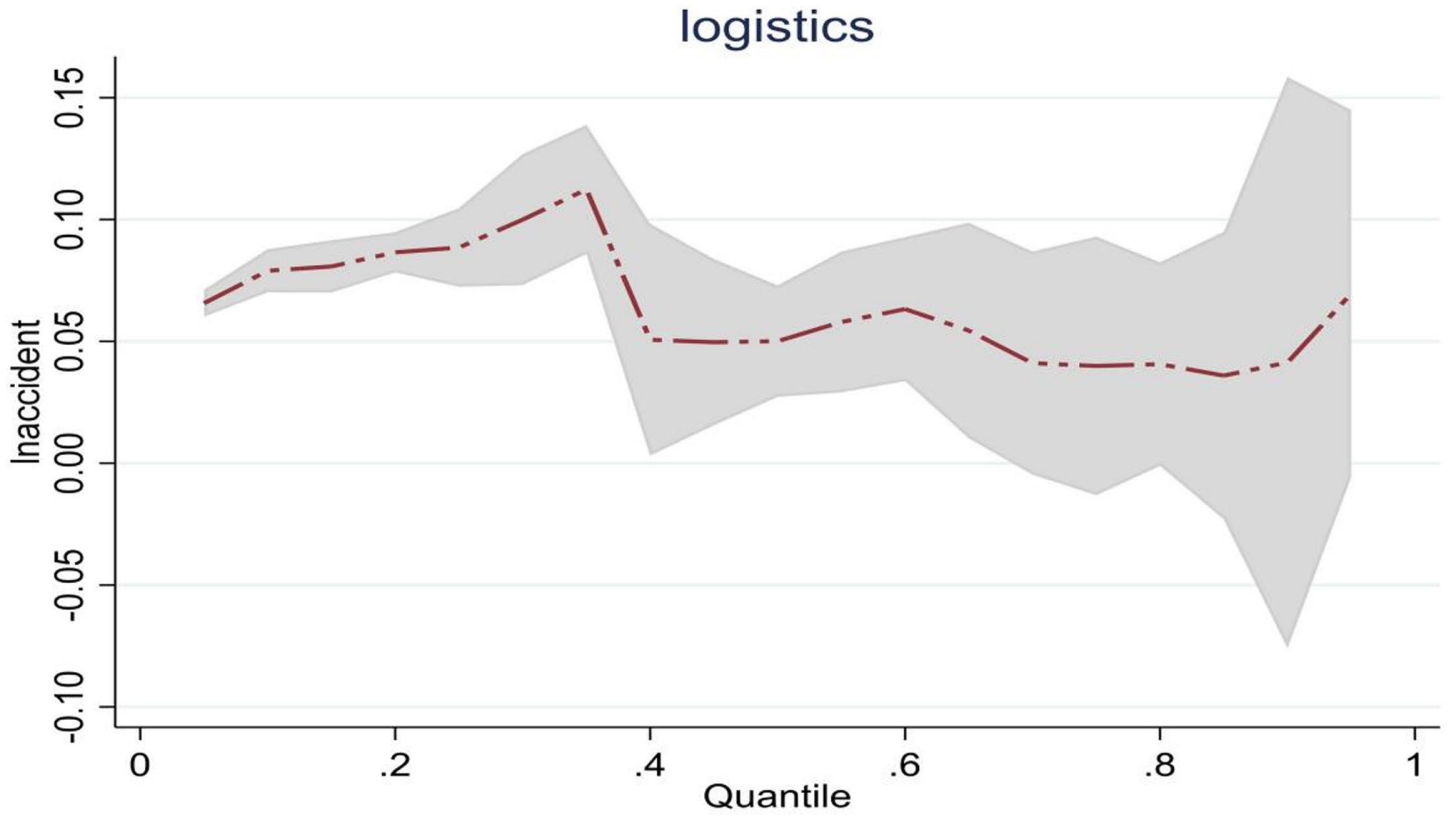
Dynamic changes of emergency logistics responsiveness in Eastern China under the impact of public emergencies.

The estimated coefficients of *lnaccident* in eastern China at quantiles 0.25, 0.5, and 0.75 are 0.089, 0.050, and 0.0399, respectively. The performance shows that as the scale of public emergencies increases, emergency logistics responsiveness shows a downtrend. As shown in Figure 5. It reflects that as the scale of public emergencies expands, due to the limitations of logistics resources, it is difficult for the emergency logistics management agencies in eastern China to continue to improve their capacity to convert general logistics into emergency logistics. Therefore, emergency logistics responsiveness in eastern China has gradually declined.

The above linear regression results show that compared with central and eastern, western regions have continuously promoted their emergency logistics responsiveness due to expanding the scale of public emergencies. However, the emergency logistics responsiveness of the eastern region has been continuously reduced due to public emergencies. In contrast, the central region was decreased first and then improved. This phenomenon may be because the central and eastern regions are facing large-scale public emergencies that have lasted for a long time and may have reached the limit of their emergency management capacities. At the same time, the limitation of logistics resources has also affected the ability of these central and eastern regions to effectively convert general logistics resources into emergency logistics, limiting the further promotion of emergency logistics responsiveness.

## 4 Identification of the causes of regional differences in China’s emergency logistics responsiveness

Previous studies have shown significant regional differences in China’s emergency logistics responsiveness. This capacity difference will weaken China’s cross-regional emergency logistics joint operations capacities if it is not optimized. Identifying the causes of regional differences in emergency logistics responsiveness will help different regions identify paths to promote emergency logistics responsiveness. This has important theoretical and practical significance for building a hierarchical, cross-regional emergency logistics rapid response system. Based on this, this paper analyzes the causes of regional differences in China’s regional emergency logistics responsiveness. The details are as follows:

### 4.1 Variable selection and description

Emergency logistics responsiveness (*emlogistics*). Based on the research goals of the paper, emergency logistics responsiveness is selected as the dependent variable, and the data comes from the measurement results in Section 3.3 of the paper. The observation period for the sample spans from 2012 to 2021.

Based on the existing literature review, this paper selects the following variables as independent variables; the data comes from the China Wind Database.^4^

#### 4.1.1 Emergency logistics technology level (technology)

Applying emergency logistics technology can effectively improve the operational efficiency of emergency logistics and thus improve emergency logistics responsiveness. However, the application of advanced technology mainly depends on whether the knowledge structure of the workforce can effectively master the technology. Therefore, when advanced technology matches the knowledge structure of the workforce, the application of emergency logistics technology can effectively promote emergency logistics responsiveness (37). To measure the level of emergency logistics technology, this paper mainly uses the number of emergency technology patent applications in the logistics industry over the years as an alternative index. It searches the CNKI patent database using the keyword “emergency.” By exploring the number of patents in various provinces (autonomous regions and municipalities) in China contained in the above keywords from 2012 to 2021, the above data are summed up as an alternative variable for the level of emergency logistics technology in different regions of China.

#### 4.1.2 Social labor input (labor)

When a public emergency occurs in society, if the scale of the public emergency is large and the existing labor supply in the logistics industry cannot effectively meet the needs of emergency logistics activities, it is necessary to introduce social labor into emergency logistics activities to ensure the stability of emergency logistics responsiveness. Effective social labor supply participation in emergency logistics activities is conducive to improving emergency logistics responsiveness (38). However, since the introduced social labor force may have different professional backgrounds, they may lack experience in emergency rescue. However, when many social labor forces are introduced into emergency logistics activities, emergency logistics activities may not be able to play a better coordination function. As a result, the driving effect on emergency logistics responsiveness will be reduced. Therefore, the driving effect of social labor input on improving emergency logistics responsiveness may have a trend of increasing and then decreasing. To measure social labor input, this paper mainly selects the total number of employed labor in China’s provinces (autonomous regions and municipalities) from 2012 to 2021 as an alternative variable (39).

#### 4.1.3 Logistics industry labor input (loglabor)

When a public emergency occurs, general logistics needs to be converted into emergency logistics, and the labor in general logistics is also converted into participants in emergency logistics activities. The more labor in general logistics is converted into emergency logistics activities, the more conducive it is to ensure the regular progress of emergency logistics activities, thereby improving emergency logistics responsiveness (40). Regarding the measurement of logistics industry labor input, this paper considers that the postal, warehousing, transportation, and handling industries account for more than 80% of the logistics industry in China. Therefore, this paper mainly selects the labor input of the postal, warehousing, transportation, and handling industries in various provinces (autonomous regions and municipalities) in China from 2012 to 2021 as an alternative variable for logistics industry labor input (41).

#### 4.1.4 Government administrative orders (order)

When the scale of public emergencies continues to expand, society needs to participate and jointly respond to public emergencies. Since emergency logistics activities are special activities that do not account for economic costs, government emergency management agencies will purchase emergency logistics resources through market-oriented behaviors to quickly improve emergency logistics responsiveness. At the same time, for government-led logistics resources, such as government-owned logistics enterprises or state-owned emergency resources, administrative orders are usually used to guide state-owned logistics resources to be quickly transformed into emergency logistics resources, thereby promoting emergency logistics responsiveness. This paper draws on the research of Bai et al. (42) and uses the proportion of state-owned economies as an index. Specifically, the ratio of state-owned fixed asset investment to total social fixed asset investment is selected as a substitute variable for government administrative orders.

#### 4.1.5 Urbanization level (Urbanization)

With the improvement of urbanization, the proportion of the urban population in the total population continues to increase. To further meet the demand of the expanding population for convenient transportation infrastructure, the government will increase investment in roads, railways, bridges, and tunnels to promote closer transportation links between cities and facilitate people’s lives and economic activities (43). For example, the construction of highways can shorten the distance between cities and promote the development of business exchanges and tourism. The construction of railways can provide a convenient way of travel and alleviate urban traffic congestion. When public emergencies occur, convenient urban transportation infrastructure is conducive to improving emergency logistics responsiveness. However, excessive urbanization will accelerate the concentration of population in cities, leading to a sharp increase in transportation demand and urban congestion. Urban congestion will have a suppressive effect on emergency logistics activities. Therefore, excessive urbanization levels may have a suppressive impact on improving emergency logistics responsiveness. As for the measurement of urbanization level, this paper selects the proportion of urban resident population as an alternative index of urbanization level (44).

#### 4.1.6 Regional logistics development level (logDGP)

Emergency logistics is the transformation of general logistics under emergency conditions. Therefore, the level of logistics development in a region determines the ability of general logistics to be transformed into emergency logistics under emergency conditions. Therefore, the higher the level of logistics development in a region, the stronger the emergency logistics responsiveness (45). This paper uses the added value of postal, warehousing, transportation, and handling industries as an alternative index of the logistics development level (46).

#### 4.1.7 Marketization level (Market)

Marketization refers to an economic operation mode that is market-oriented and based on financial activities. It realizes resource allocation and economic development through market mechanisms such as competition, supply and demand relations, and price mechanisms (47). However, in the case of public emergencies, emergency logistics activities are weak economic activities that do not consider costs. Therefore, in the case of excessive marketization, if the commercial interests of emergency logistics activities are overemphasized, the interests of public safety will be ignored. Thus, improving the marketization level has a suppressive effect on improving emergency logistics responsiveness (14). This paper selects the “China Marketization Index Report” marketization index as an alternative index.

#### 4.1.8 Digital level (Digital)

The improvement of the digital level can promote the matching of emergency logistics supply and demand information, coordinate various modes of transportation, optimize logistics operations, and improve emergency logistics responsiveness (48). For the measurement of the digital level, based on the existing research on the definition of digitalization, a digital index system including digital infrastructure, digital scale, digital industry, and digital innovation is constructed, and the entropy weight TOPSIS method of panel data is used to measure the digital economy (49).

Based on the above variables and their influencing mechanisms, taking into account the possible regulatory effects of government administrative orders and marketization levels on social labor input and logistics industry labor input, the specific mechanism analysis is as follows:

The regulatory role of government administrative orders and marketization on social labor and logistics labor input. When a public emergency occurs, the government emergency management department needs to immediately transform general logistics into emergency logistics so that the labor of the logistics industry will be transformed into the labor required for emergency logistics activities. Since China’s logistics enterprises are divided into two types, state-owned enterprises, and private enterprises, administrative orders can be used to mobilize the labor of state-owned logistics enterprises to transform into the labor force in emergency logistics activities. At the same time, the labor of private logistics enterprises can be purchased through market-based price mechanisms to encourage them to transform into the labor required for emergency logistics activities. Therefore, government administrative orders and marketization have a regulatory role on the labor input of the logistics industry, thereby exerting an impact on emergency logistics responsiveness. However, after the labor of the logistics industry is transformed into the labor required for emergency logistics activities, it is necessary to introduce social labor into emergency logistics activities if it fails to achieve the goals that emergency logistics should achieve. Similarly, the emergency management department can also guide social labor in participating in emergency logistics through government administrative orders and market-based price mechanisms, affecting emergency logistics responsiveness.

### 4.2 Basic data statistics and description

Table 11 summarizes the data processed in the previous paper. According to the summary results, from the standard deviation of each variable, the standard deviation of *labor* is the largest, and the standard deviation of *order* is the smallest. To further measure the fluctuation of data differences, this paper uses the mean and standard deviation to calculate the coefficient of variation of the data. From the coefficient of variation, *Digital* has the largest coefficient of variation, while *Market* has the smallest coefficient of variation. This indicates that the difference between the data of the variable *Digital* is the largest, while the difference between the data of the variable *Market* is the smallest.

**Table 11:** Statistical characteristics of relevant data.

Variable name	Number of observations	Average value	Standard deviation	Coefficient of variation	Minimum	Maximum
*Emlogistics*	270	0.187	0.0936	0.5005	0.044	0.595
*Technology*	270	192.844	274.99	1.425	1	2058
*Labor*	270	1624.49	1292.29	0.795	130.73	7962.92
*Loglabor*	270	27.25	16.832	0.617	3.4	86.41
*Order*	270	0.010	0.0059	0.59	0.00122	0.044
*Urbanization*	270	59.7	11.86	0.198	36.3	89.6
*logGDP*	270	1185.36	836.77	0.705	71.87	3855.78
*Market*	270	8.03	1.859	0.231	3.359	11.934
*Digital*	270	0.122	0.111	0.909	0.013	0.725

Original data source: wind database, sample observation period 2012–2021.

### 4.3 Construction of econometric model

This study selects emergency logistics responsiveness as the dependent variable based on the mechanism relationship analysis between the above variables. Based on identifying the causes of regional emergency logistics responsiveness, emergency logistics technology, social labor input, logistics industry labor input, government administrative orders, urbanization level, regional logistics development level, marketization level, and digitalization level are used as dependent variables. The mechanism relationship between the above variables is verified by constructing a panel data regression model. The model is built as shown in Formula 4-1:


(4-1) $$\begin{array}{l} \mathrm{lnemlogistic}s_{\mathrm{it}}=c+\beta_{1}\mathrm{lntechnolog}y_{\mathrm{it}}+\beta_{2}\mathrm{lnlabo}r_{\mathrm{it}}\\ +\beta_{3}\mathrm{lnloglabo}r_{\mathrm{it}}+\beta_{4}l\mathrm{norde}r_{\mathrm{it}}+\beta_{5}\mathrm{lnUranizatio}n_{\mathrm{it}}\\ +\beta_{6}\mathrm{lnlogGD}P_{\mathrm{it}}+\beta_{7}\mathrm{lnMarke}t_{\mathrm{it}}+\mathrm{lnDigita}l_{\mathrm{it}}+\mu_{\mathrm{it}} \end{array}$$

Based on the above analysis of the impact of social labor input on emergency logistics responsiveness. To verify the nonlinear effect of social labor input on emergency logistics responsiveness, this paper constructs a panel quantile regression model as shown in Formula 4-2:


(4-2) $$\mathrm{lnemlogistics}=\alpha_{1q}+b_{2q}\mathrm{lnlabo}r_{tq}+\mu_{tq}$$

In addition, to verify the regulatory effect of government administrative orders and marketization on social labor and logistics industry labor input, this paper constructs the panel quantile regression model shown in Formula 4-3:


(4-3) $$\begin{array}{l} \mathrm{lnemlogistic}s_{\mathrm{it}}=c+\beta_{1}\mathrm{lntechnolog}y_{\mathrm{it}}+\beta_{2}\mathrm{lnlabo}r_{\mathrm{it}}\\ +\beta_{3}\mathrm{lnloglabo}r_{\mathrm{it}}+\beta_{4}\mathrm{lnorde}r_{\mathrm{it}}+\beta_{5}\mathrm{lnUranizatio}n_{\mathrm{it}}\\ +\beta_{6}\mathrm{lnlogGD}P_{\mathrm{it}}+\beta_{7}\mathrm{lnMarke}t_{\mathrm{it}}+\beta_{8}\mathrm{lnDigita}l_{\mathrm{it}}\\ +\beta_{9}\mathrm{lnorde}r_{\mathrm{it}}*\mathrm{lnlabo}r_{\mathrm{it}}+\beta_{10}ln\mathrm{orde}r_{\mathrm{it}}*\mathrm{lnloglabo}r_{\mathrm{it}}\\ +\beta_{11}\mathrm{lnMarke}r_{\mathrm{it}}*\mathrm{lnlabo}r_{\mathrm{it}}+\beta_{12}\mathrm{lnMarke}r_{\mathrm{it}}\\ {}*\mathrm{lnloglabo}r_{\mathrm{it}}+\mu_{\mathrm{it}} \end{array}$$

In Model (4-1), Model (4-2), and Model (4-3), *lnemlogistics* stands for the emergency logistics responsiveness; *lntechnology* stands for the level of emergency logistics technology; *lnlabor* stands for social labor input; *lnloglabor* stands for the labor input of the logistics industry; *lnorder* stands for government administrative orders; *lnUrbanization* stands for the level of urbanization; *lnlogGDP* stands for the level of regional logistics development; *lnMarket* stands for the level of marketization; *lnDigital* stands for the level of digitalization; the interaction term lnorder*lnlabor stands for the regulatory effect of government administrative orders on social labor input; lnorder*lnloglabor stands for the regulatory impact of government administrative orders on labor input of the logistics industry; lnMarketr*lnlabor stands for the regulatory effect of marketization level on social labor input; lnMarketr*lnloglabor stands for the regulatory impact of marketization level on labor input of the logistics industry. $\mu_{\mathrm{it}}$ stands for a random disturbance term; *it* stands for the sample of Chinese provinces from 2012 to 2021.

### 4.4 Analysis of empirical results

#### 4.4.1 Examination of the critical causes of regional differences in China’s emergency logistics responsiveness

The linear regression results in Table 12 show that the national sample observation’s regression coefficient of *lntechnology* is insignificant. The possible reason is that the degree of application of emergency logistics technology is not high, or there may be a mismatch between the labor knowledge structure and emergency logistics technology. The *lnlabor* regression coefficient is 0.2252 and significant, while the *lnloglabor* coefficient is insignificant. It shows that increasing social labor input can improve emergency logistics responsiveness. It also indirectly reflects that when public emergencies occur, the labor force in the logistics industry is insufficient to be converted into the labor force required for emergency logistics, resulting in the inability of the existing logistics industry’s labor input to meet the needs of emergency logistics activities effectively. The *lnorder* regression coefficient is −0.0307 and is significant, indicating that the administrative order method cannot effectively promote emergency logistics responsiveness but instead inhibits emergency logistics responsiveness. The possible reason is that administrative orders lead to inefficient resource allocation. Emergency logistics requires a high degree of flexibility and rapid response, while administrative orders are usually based on fixed instructions and processes, which may not be able to adjust in time or meet the needs of different regions and different types of emergency events. The *lnUrbanization* regression coefficient is 0.6865, indicating that increasing urbanization can improve emergency logistics responsiveness. For the whole country, increasing urbanization can effectively promote the optimization of urban infrastructure and thereby improve emergency logistics responsiveness. The *lnlogGDP* regression coefficient is 0.0389 and significant, indicating that improving the logistics development level can promote emergency logistics responsiveness. The *lnMarket* regression coefficient is −0.2078 and is significant, indicating that the improvement of marketization level has an inhibitory effect on promoting emergency logistics responsiveness. The main reason may be that emergency logistics activities are weak economic activities regardless of cost, and the commercial nature of China’s emergency logistics activities benefits are overemphasized, which is harmful to improving emergency logistics responsiveness. The *lnDigital* regression coefficient is 0.09476 and significant, indicating that increasing the digitalization level can improve emergency logistics responsiveness. Digital technology has improved the efficiency of information flow and coordination. Through real-time data sharing and intelligent scheduling systems, digitalization enables all parties to quickly obtain accurate logistics information, thereby optimizing transportation routes, scheduling resources, and improving response speed.

**Table 12:** Factors affecting regional differences in China’s emergency logistics responsiveness and their effects.

Variable	National	Eastern	Central	Western
	*lnemlogistics*	*lnemlogistics*	*lnemlogistics*	*lnemlogistics*
*lntechnology*	−0.0220 (0.01619)	−0.0376 (0.04591)	0.6422*** (0.3626)	−0.021 (0.0146)
*lnlabor*	0.2252*** (0.0509)	0.24573** (0.1216)	−4.141** (1.978)	0.0443 (0.0657)
*lnloglabor*	−0.06194 (0.0488)	−0.1498 (0.1107)	−0.4161 (0.9944)	0.08715 (0.0567)
*lnorder*	−0.0307*** (0.01527)	0.0372 (0.04295)	0.3284 (0.2927)	−0.03554* (0.0205)
*lnUrbanization*	0.6865*** (0.1775)	0.8256* (0.4369)	−4.290** (2.065)	0.8331*** (0.2657)
*lnlogGDP*	0.0389* (0.0300)	−0.1057 (0.1038)	−0.2675 (0.8788)	0.1142*** (0.0297)
*lnMarket*	−0.2078*** (0.0725)	0.0533 (0.2113)	3.0152 (2.192)	−0.2837*** (0.0800)
*lnDigital*	0.09476*** (0.0309)	0.2136*** (0.0782)	2.110** (0.9427)	0.0773*** (0.0353)
*C*	−5.6535*** (0.8068)	−4.828*** (1.776)	30.233* (17.26)	−6.016*** (1.0114)
*F*-statistic	70.83***	28.99***	11.64***	46.29***
*R* ^2^	0.6595	0.4152	0.1698	0.5529
Regression method	FE	FE	FE	FE

*** indicates significant at the 1% level; ** indicates significant at the 5% level; * indicates significant at the 10% level.

The linear regression results of regional samples (Table 12) show that for the western and eastern regions, the regression coefficient of *lntechnology* is insignificant, and only the coefficient of *lntechnology* in the central region is 0.6422, which is significant. It shows that the degree of application of emergency logistics technology in the western and eastern regions is not high, or there may be a mismatch between the labor knowledge structure and emergency logistics technology. In contrast, the degree of application of emergency logistics technology in the central region is relatively high. The regression coefficient of *lnlabor* in the eastern region is 0.24573 and significant, the coefficient in the central region is −4.141 and significant, and the coefficient in the western region is 0.0443 but not significant. This reflects that introducing social labor in the eastern region can promote emergency logistics responsiveness. In contrast, introducing social labor in the central region may inhibit emergency logistics responsiveness due to low professional quality. However, the western region may be unable to effectively promote emergency logistics responsiveness due to insufficient introduced social labor. The *lnloglabor* regression coefficients in the western, central, and eastern regions are insignificant. The regression coefficient of *lnorder* in the central and eastern regions is insignificant, while the coefficient in the western region is −0.03554. It shows that adopting administrative orders in the central and eastern regions cannot promote emergency logistics responsiveness. However, administrative orders in the western region have inhibited emergency logistics responsiveness. The regression coefficients of *lnUrbanization* in the western and eastern regions are 0.8256 and 0.8331, respectively, and are significant, while the coefficient in the central region is −4.290 and is significant. It reflects that the urbanization level in the western and eastern regions can effectively promote the optimization of urban infrastructure and improve emergency logistics responsiveness. However, although the urbanization rate in the central region has increased, excessive urbanization may accelerate the concentration of the population in cities. This leads to a sharp increase in transportation demand and prominent urban congestion problems, thereby inhibiting emergency logistics responsiveness. The regression coefficients of *lnMarket* in the central and eastern regions are insignificant, but the coefficient in the western region is −0.2837. From the side, it reflects that the western region attaches more importance to commercial interests in emergency logistics activities, so the improvement of marketization level has an inhibitory effect on improving emergency logistics responsiveness. The regression coefficients of *lnDigital* in the western, central, and eastern regions are 0.2136, 2.110, and 0.0773, respectively. They are all significant, indicating that improving digitalization levels in the western, central, and eastern regions can effectively improve emergency logistics responsiveness. Digitalization has the most substantial promoting effect in the central region and the weakest in the western region.

**Figure 6:**
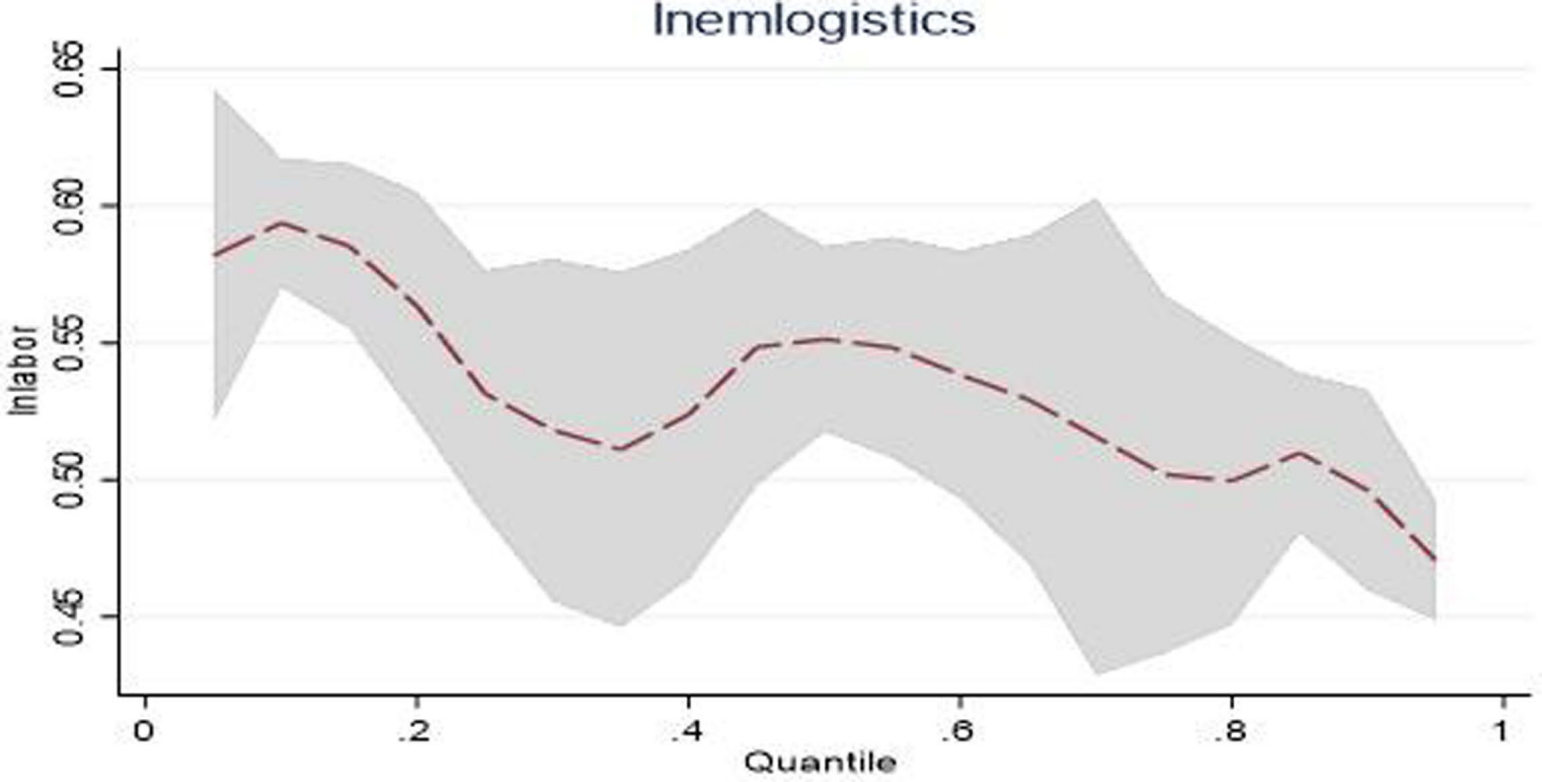
The impact of the introduction of national social labor on emergency logistics responsiveness.

**Figure 7:**
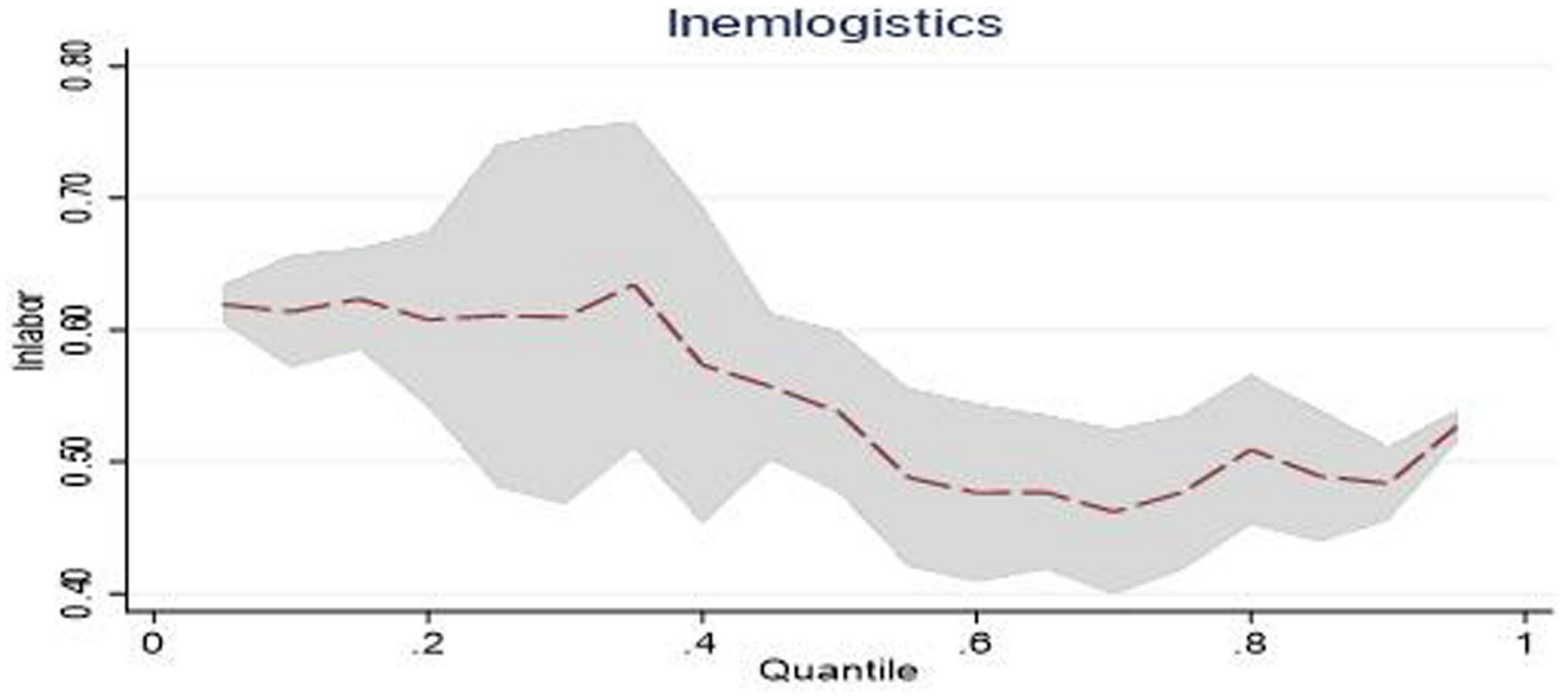
The impact of social labor introduction in Eastern China on emergency logistics responsiveness.

**Figure 8:**
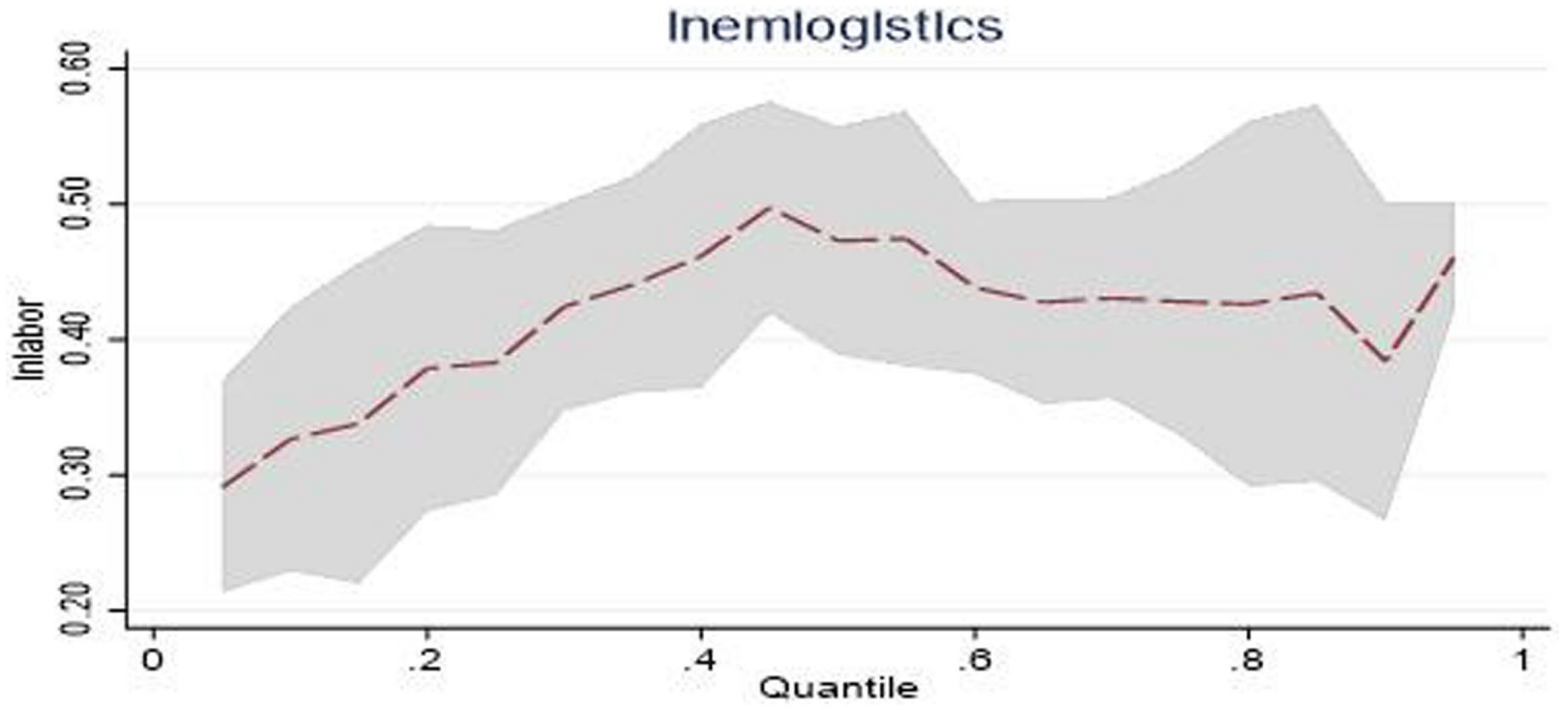
The impact of social labor introduction in Western China on emergency logistics responsiveness.

Further, combined with the mechanism analysis of this paper, the nonlinear impact of social labor input was tested. The results of linear regression are shown in Table 13. Observed from the linear regression results of the national sample, the estimated coefficients of *lnlabor* at the quantiles 0.25, 0.5, and 0.75 are 0.5315, 0.5513, and 0.5020, respectively, and they are all significant. From the trend observation, as the level of social labor input continues to increase, its role in improving emergency logistics responsiveness first increases and then decreases (Figure 6). It shows that for the whole country, when the labor force in the logistics industry cannot meet the demand for emergency logistics activities, introducing an appropriate amount of social labor as a supplement can effectively promote emergency logistics responsiveness. However, since the social labor force lacks the necessary professional capacities and experience in emergency logistics, continuously increasing the scale of social labor introduction may not be conducive to promoting the coordination of emergency logistics activities. Instead, it will reduce emergency logistics responsiveness. Observed from the linear regression results of the samples in the eastern region, the estimated coefficients of *lnlabor* at the quantiles 0.25, 0.5, and 0.75 are 0.610, 0.5373, and 0.4769, respectively, and they are all significant. From the trend observation, as the level of social labor input continues to increase, its role in improving emergency logistics responsiveness continues to decline. It reflects that introducing a social labor force to participate in emergency logistics activities in the eastern region cannot be used as an effective labor force supplement. The emergency professional capacities of the introduced social labor force are not suitable for emergency logistics activities, which leads to a continuous increase in the scale of social labor input. Its role in improving emergency logistics responsiveness continues to decline (Figure 7). Observed from the linear regression results of the central region, its estimated coefficients at quantiles 0.25, 0.5, and 0.75 are not significant. The possible reason is that the central region’s logistics infrastructure and emergency logistics system are imperfect, making it difficult to effectively transform labor into the actual driving force of emergency logistics. Observed from the linear regression results in the western region, the estimated coefficients of *lnlabor* at quantiles 0.25, 0.5, and 0.75 are 0.382, 0.472, and 0.428, respectively, and they are all significant. From the trend observation, as the scale of social labor introduction increases, its driving role in improving emergency logistics responsiveness increases and decreases (Figure 8). The possible reason is that in the western region, introducing social labor can effectively supplement the missing labor in emergency logistics activities, thereby improving emergency logistics responsiveness. However, as the scale of introduced labor continues to expand, the quality and efficiency of labor may decline due to problems in management, training and coordination, resulting in a gradual weakening of the role of improving emergency logistics responsiveness.

**Table 13:** Test of the nonlinear impact of social labor input on emergency logistics responsiveness.

Sample	Variable	QR_0.25	QR_0.5	QR_0.75
National	*lnlabor*	0.5315*** (0.0315)	0.5513*** (0.0254)	0.5020*** (0.0310)
	Intercept (C)	−5.779*** (0.2255)	−5.687*** (0.1819)	−5.127*** (0.222)
Eastern	*lnlabor*	0.610*** (0.0498)	0.5373*** (0.053)	0.4769*** (0.0307)
	Intercept (C)	−6.3693*** (0.3763)	−5.548*** (0.4051)	−4.919*** (0.232)
Central	*lnlabor*	−0.289 (1.161)	−0.108 (0.467)	0.1007 (0.6985)
	Intercept (C)	−2.875 (1.761)	−2.387*** (0.708)	−1.560 (1.059)
Western	*lnlabor*	0.382*** (0.052)	0.472*** (0.040)	0.428*** (0.051)
	Intercept (C)	−4.843*** (0.347)	−5.218*** (0.272)	−4.774*** (0.3448)

*** indicates significant at the 1% level; ** indicates significant at the 5% level; * indicates significant at the 10% level.

#### 4.4.2 Administrative orders and marketization levels regulatory mechanism test

Combined with the research objectives, this paper examines the regulatory mechanisms of administrative orders and marketization levels on social labor and logistics industry labor. The results are shown in Table 14. As observed from the national sample linear regression results, the regression coefficient of lnorder*lnlabor is −0.0886, which is significant. It shows that through the regulation of administrative orders, the introduction of social labor has an inhibitory effect on improving emergency logistics responsiveness. The regression coefficient of lnMarketr*lnlabor is 0.2406 and is significant. It shows that adjusting the marketization level positively promotes emergency logistics responsiveness by introducing social labor. The above results reflect that when public emergencies occur, for the whole country, government administrative orders cannot effectively guide the social workforce to participate actively in emergency logistics activities. Instead, they will inhibit improving emergency logistics responsiveness due to non-motivation. On the other hand, the role of marketization and the use of price mechanisms to introduce social labor can enhance the enthusiasm of social labor to participate in emergency activities, thereby effectively improving emergency logistics responsiveness. The regression coefficient of lnorder*lnloglabor is 0.1409 and significant, but the coefficient of lnMarketr*lnloglabor is insignificant. It shows that for the entire country, administrative orders can be used to convert the labor force in the logistics industry into a labor force for emergency logistics activities. However, the use of market-based price mechanisms will not be effective. The possible reason is that the labor force in the logistics industry usually has certain professional skills and strong organization, especially in state-owned enterprises, where employees usually respond directly to government instructions. Through administrative orders, the government can quickly mobilize the labor force in the logistics industry to participate in emergency logistics activities. Although the market mechanism can attract social labor to participate in emergency logistics activities through price incentives, for the labor force already working in the logistics industry, pure market incentives may not be able to change their original work arrangements or functional transformation quickly.

**Table 14:** Administrative orders and marketization levels regulatory mechanism test.

Variable	National	Eastern	Central	Western
	*lnemlogistics*	*lnemlogistics*	*lnemlogistics*	*lnemlogistics*
*lntechnology*	−0.0245** (0.0137)	0.0090 (0.0356)	0.3238 (0.4467)	−0.0173 (0.0129)
*lnlabor*	−0.875*** (0.2076)	−2.3763*** (0.6685)	5,007 (200246)	−0.6384** (0.2576)
*lnloglabor*	−1.494*** (0.3261)	−2.0755*** (0.8440)	15,183 (13.426)	−0.9590* (0.5264)
*lnorder*	0.266*** (0.0696)	0.57867*** (0.1399)	0.1055 (0.8629)	0.1872** (0.0978)
*lnUrbanization*	0.6459*** (0.1481)	1.3765*** (0.3509)	−2.469 (2.4636)	0.6155*** (0.2228)
*lnlogGDP*	−0.0272 (0.0259)	−0.10229 (0.0824)	−0.2621 (0.9464)	0.0320 (0.0276)
*lnMarket*	−1.722*** (0.3605)	−5.205*** (1.1533)	30.682* (18.104)	−1.7181*** (0.4538)
*lnDigital*	0.1606*** (0.0270)	0.1606*** (0.0655)	1.4397* (1.0529)	0.1482*** (0.0311)
lnorder*lnlabor	−0.0886*** (0.0216)	−0.1741*** (0.0438)	0.0717 (0.2410)	−0.0694** (0.0339)
lnorder*lnloglabor	0.1409*** (0.0306)	0.1943*** (0.0443)	−2.2776 (3.2784)	0.0728 (0.0650)
lnMarketr*lnlabor	0.2406*** (0.0878)	0.7503*** (0.2715)	−0.2750 (9.0399)	0.2009** (0.0982)
lnMarketr*lnloglabor	0.0403 (0.1203)	−0.1155 (0.4111)	−9.059* (5.653)	0.1236 (0.1247)
C	3.6465*** (1.241)	13.470*** (3.0942)	−31.813 (45.936)	1.295 (1.840)
*F*-statistic	77.82***	39.26***	11.40***	50.44***
*R* ^2^	0.2278	0.2144	0.2189	0.1277
Regression methods	FE	FE	FE	FE

*** indicates significant at the 1% level; ** indicates significant at the 5% level; * indicates significant at the 10% level.

From the linear regression results of sub-regional samples, the regression coefficients of lnorder*lnlabor in the western and eastern regions are −0.1741 and − 0.0694, respectively, and both are significant. In contrast, the regression coefficients of lnMarketr*lnlabor are 0.7503 and 0.2009, respectively, which are significant. It reflects that government administrative orders in the western and eastern regions cannot effectively guide the social labor force to participate actively in emergency logistics activities. On the contrary, it will inhibit improving emergency logistics responsiveness for non-motivation reasons. Through marketization, Using the price mechanism to introduce social labor can enhance the enthusiasm of social labor to participate in emergency activities, thereby effectively improving emergency logistics responsiveness. However, it is not significant in the central region or above. This may be because the emergency logistics system in the central region is relatively weak, and the participation of the social labor force is low, resulting in a weak regulatory effect. However, only the regression coefficient of lnorder*lnloglabor is significant in the eastern region, not the western and central regions. It shows that government administrative orders can effectively enhance the transformation of the labor force in the logistics industry into a labor force for emergency logistics activities and effectively improve emergency logistics responsiveness. This may be because the eastern region has a higher level of economic development, complete logistics infrastructure and an efficient government execution system. Government administrative orders enable professional labor to be quickly transformed into labor for emergency logistics activities, improving emergency logistics responsiveness. However, the regression coefficients of lnMarketr*lnloglabor in the western, central, and eastern regions are insignificant.

## 5 Discussion

This paper analyses the differences in emergency logistics responsiveness in China and their causes. Firstly, existing studies have a single evaluation index for emergency logistics responsiveness, and most methods tend to be subjective assignment methods or mathematical analysis methods. This study expands the research on emergency logistics responsiveness evaluation. Based on the constituent factors of emergency logistics responsiveness, this study constructs a comprehensive index system for emergency logistics responsiveness. The entropy weight TOPSIS method measures the emergency logistics responsiveness of different regions in China. In addition, research on emergency logistics responsiveness tends to be evaluated in a single dimension, which limits the diversity of evaluation indexes. This paper proposes a comprehensive evaluation framework that includes multiple dimensions. Compared with existing literature, this framework can more comprehensively reflect the nature and characteristics of emergency logistics responsiveness and provide a more comprehensive perspective for evaluation. The study found that the emergency logistics responsiveness of China’s western, central, and eastern regions showed a steady upward tendency. The emergency logistics responsiveness of the eastern region is greater than that of the western and central regions.

Secondly, this paper further explores the definition, classification, and impact of public emergencies. It points out that existing research is insufficient in the quantitative analysis of the impact of these events. In order to fill this gap, this paper uses a panel quantile regression model to quantitatively analyze the specific effect of public emergencies on emergency logistics responsiveness. It adds a new dimension to the relevant discussion. The results show that China’s emergency management agencies can effectively mobilize and utilize general logistics resources to respond to emergencies. When responding to public emergencies, practical experience can be continuously summarized, and emergency logistics plans can be optimized. It effectively promotes emergency logistics responsiveness. However, from the trend analysis, when the scale of the event reaches a certain level, its positive impact on emergency logistics responsiveness begins to weaken. It may be related to the increased complexity of the event and the limitation of logistics resources, which makes it difficult for emergency management agencies to enhance their emergency logistics responsiveness further.

From the perspective of regional differences: The scale of public emergencies in the western region is proportional to the emergency logistics responsiveness. In contrast, under the influence of public emergencies, the emergency logistics responsiveness of the eastern region has been declining, while the central region has experienced a decline and then an increase. This difference may be because the scale of public emergencies faced by the central and eastern regions is much larger than that of the western region. As the scale of public emergencies in central and eastern regions continues to expand, the emergency management capacity of these regions may have reached its limit. In addition, the limitation of logistics resources also hinders the effective transformation of general logistics to emergency logistics. Thus, it is impossible to drive the emergency logistics responsiveness to continue improving.

Finally, the study on regional differences in the development of the logistics industry has been expanded, overcoming the fact that existing studies mainly focus on the study of regional differences in the development of the logistics industry. However, the analysis of regional differences in emergency logistics responsiveness has been neglected. Due to differences in the operation, organization, and participation of social and emergency logistics, there are also regional differences in the emergency logistics responsiveness of different regions. Such differences may constrain the cross-regional emergency logistics joint action capacities. However, existing studies have paid less attention to exploring this problem, and the causes of differences in regional emergency logistics responsiveness are rarely involved. Therefore, this paper uses the Dagum Gini coefficient method to explore the differences in China’s regional emergency logistics responsiveness and analyzes the sources of the differences. The study found that the regional differences in China’s emergency logistics responsiveness are expanding, and they are mainly due to differences between regions.

The research on the causes of regional differences in emergency logistics responsiveness found that:

Firstly, the application level of emergency logistics technology in the western and eastern regions is not high, or there is a phenomenon that the knowledge structure of the labor force may not match the emergency logistics technology. In contrast, the application level of emergency logistics technology in the central region is relatively high. Introducing social labor in the eastern region can promote emergency logistics responsiveness. In contrast, when introducing social labor in the central region, emergency logistics responsiveness may be inhibited due to the low quality of the professionals. However, the western region may not be able to effectively promote emergency logistics responsiveness due to the lack of social labor introduced. The central and eastern regions cannot facilitate emergency logistics responsiveness through administrative orders. In contrast, the western region has inhibited emergency logistics responsiveness through administrative orders. The urbanization level in the western and eastern regions can effectively promote the optimization of urban infrastructure and thus improve emergency logistics responsiveness. However, excessive urbanization in the central region may accelerate city population gathering. It leads to a sharp increase in traffic demand and more prominent urban congestion problems, inhibiting emergency logistics responsiveness. Commercial interests are more valued in the western region’s emergency logistics activities, so the marketization level inhibits emergency logistics responsiveness. The digitalization level in the western, central, and eastern regions can effectively promote emergency logistics responsiveness. The promotion effect of digitalization on the central region is the strongest, and the western region is the weakest. The introduction of social labor to participate in emergency logistics activities in the eastern region cannot serve as an effective labor supplement, resulting in a continuous decline in the role of social labor input in improving emergency logistics responsiveness. In the western region, as the scale of social labor introduction increases, its driving effect on improving emergency logistics responsiveness increases and decreases.

Secondly, the western and eastern regions cannot effectively guide the social labor force to participate actively in emergency logistics through government administrative orders. Instead, it will have a suppressive effect on improving emergency logistics responsiveness for non-active reasons. Through marketization, the social labor force can be introduced through the price mechanism. It can enhance the enthusiasm of the social labor force to participate in emergency activities, thereby effectively improving emergency logistics responsiveness. The administrative orders of the eastern region government can effectively enhance the transformation of the logistics industry labor force into the labor force for emergency logistics activities. Therefore, it effectively promotes emergency logistics responsiveness.

From a practical perspective, China is one of the largest countries in the world, with a vast territory spanning north and south, east and west. Different geographical and climatic conditions have led to various types of public emergencies faced by China. It is essential to clarify the regional differences and causes of China’s emergency logistics responsiveness. This is of great reference significance for different countries worldwide to improve their emergency logistics responsiveness. In addition, it is adequate to identify the critical factors for improving China’s emergency logistics responsiveness under the impact of public emergencies. This will promote joint emergency logistics rescue cooperation between China and other countries worldwide and play an essential role in stabilizing global economic development. It will also help promote China and the international community to jointly respond to global challenges, such as public emergencies, and maintain international order.

## 6 Conclusion and implications

### 6.1 Research conclusions

1. Observation from the measurement results of China’s emergency logistics responsiveness: The emergency logistics responsiveness of the western, central, and eastern regions has shown a steady upward trend. The emergency logistics responsiveness of the eastern region is greater than that of the western and central regions.
2. Observation from the differences and trends of China’s regional emergency logistics responsiveness: The study revealed significant differences in China’s emergency logistics responsiveness among different regions. These differences are mainly caused by uneven development between regions. China’s emergency management agencies have shown significant efficiency in converting general logistics resources into emergency logistics, thus improving emergency responsiveness. However, when faced with large-scale public emergencies, this transformation capacity seems to have reached its limit, causing emergency logistics responsiveness to decline. From a regional perspective, compared with the central and eastern regions, the western region’s emergency logistics responsiveness has been enhanced due to the expansion of the scale of public emergencies. However, in the eastern region, due to the continued impact of public emergencies, the responsiveness of emergency logistics has shown a downward trend. The central region has experienced a process of first falling and then rising.
3. The causes of regional differences in China’s emergency logistics responsiveness were analyzed. The innovative conclusions are as follows:

#### 6.1.1 National sample regression results

Firstly, the level of emergency logistics technology cannot effectively promote emergency logistics responsiveness. However, the level of labor input in the logistics industry is insignificant, indicating that when a public emergency occurs, the labor force in the logistics industry is converted into insufficient labor required for emergency logistics. This results in the labor input in the existing logistics industry being unable to effectively meet the needs of emergency logistics activities. The administrative order method inhibits emergency logistics responsiveness. Improving the marketization level has an inhibitory effect on improving emergency logistics responsiveness. The main reason may be that emergency logistics activities are weak economic activities regardless of cost. The commercial interests of China’s emergency logistics activities may be overemphasized, which is not conducive to promoting emergency logistics responsiveness. The level of social labor input, urbanization level, logistics development level, and digitalization level can effectively promote emergency logistics responsiveness.

Secondly, when the logistics industry’s labor force cannot meet emergency logistics activities’ needs, introducing an appropriate amount of social labor as a supplement can effectively promote emergency logistics responsiveness. However, since social labor lacks the necessary professional capacities and experience in emergency logistics, continuously increasing the scale of social labor introduction may not be conducive to improving the coordination of emergency logistics activities. However, it will reduce emergency logistics responsiveness.

Finally, when a public emergency occurs, the government’s administrative orders cannot effectively guide the social labor force to participate actively in emergency logistics activities. Instead, the lack of enthusiasm will have a suppressive effect on promoting emergency logistics responsiveness. On the contrary, through marketization, introducing social labor through the price mechanism can enhance the excitement of social labor to participate in emergency activities, thereby effectively improving emergency logistics responsiveness. However, administrative orders can transform the logistics industry labor into the labor force for emergency logistics activities, while the market-based price mechanism cannot produce any effect.

#### 6.1.2 Regional sample regression results

Firstly, the application level of emergency logistics technology in the western and eastern regions is not high, or there is a phenomenon that the knowledge structure of the labor force may not match the emergency logistics technology. In contrast, the application level of emergency logistics technology in the central region is relatively high. Introducing social labor in the eastern region can promote emergency logistics responsiveness. In contrast, when introducing social labor in the central region, emergency logistics responsiveness may be inhibited due to the low quality of the professionals. However, the western region may not be able to effectively promote emergency logistics responsiveness due to the lack of social labor introduced. The central and eastern regions cannot facilitate emergency logistics responsiveness through administrative orders. In contrast, the western region has inhibited emergency logistics responsiveness through administrative orders. The urbanization level in the western and eastern regions can effectively promote the optimization of urban infrastructure and thus improve emergency logistics responsiveness. However, excessive urbanization in the central region may accelerate city population gathering. It leads to a sharp increase in traffic demand and more prominent urban congestion problems, inhibiting the improvement of emergency logistics responsiveness. Commercial interests are more valued in the western region’s emergency logistics activities, so the marketization level inhibits emergency logistics responsiveness. The digitalization level in the western, central, and eastern regions can effectively promote emergency logistics responsiveness. The promotion effect of digitalization on the central region is the strongest, and the western region is the weakest. The introduction of social labor to participate in emergency logistics activities in the eastern region cannot serve as an effective labor supplement, resulting in a continuous decline in the role of social labor input in promoting emergency logistics responsiveness. In the western region, as the scale of social labor introduction increases, its driving effect on improving emergency logistics responsiveness increases and decreases.

Secondly, the western and eastern regions cannot effectively guide the social labor force to participate actively in emergency logistics through government administrative orders. Instead, it will have a suppressive effect on improving emergency logistics responsiveness for non-active reasons. Through marketization, the social labor force can be introduced through the price mechanism. It can enhance the enthusiasm of the social labor force to participate in emergency activities, thereby effectively improving emergency logistics responsiveness. The administrative orders of the eastern region government can effectively enhance the transformation of the logistics industry labor force into the labor force for emergency logistics activities. Therefore, it effectively promotes emergency logistics responsiveness.

### 6.2 Research implications

Based on the above research conclusions, this paper obtains the following implications:

Firstly, China’s emergency logistics responsiveness is rising, but the overall level is still low. Therefore, governments at all levels should gradually improve emergency logistics regulations or standards and establish a permanent agency for unified emergency logistics management. Clarify the social responsibilities of emergency management and logistics functional agencies. Rationally allocate and improve emergency material production capacity and layout, optimize the balance between supply and demand of emergency materials, and achieve efficient transportation and adequate supervision. Make it play the role of critical nodes, essential platforms, and hub services in the national emergency logistics network to ensure the efficient operation of the emergency system.

Secondly, China’s emergency logistics responsiveness is unevenly distributed, with the east superior and the west inferior, and the regional gap is also expanding. How to continue to improve emergency logistics responsiveness, solve the uneven distribution situation, and curb the expansion of differences are the core issues that need to be addressed. Therefore, regional differences should be noted, especially the development differences between the three major economic regions. Relying on the existing comprehensive three-dimensional transportation network and logistics system, and based on the characteristics of emergencies in each region, each region should improve the transportation network layout and plan and build emergency logistics hubs. Continuously strengthen regional exchanges and cooperation to narrow the gap between regions in emergency logistics responsiveness.

Thirdly, unified data sharing, digitally visible emergency logistics information release, and a command and coordination platform should be built to achieve real-time control, sharing, and communication of emergency logistics resources on the platform. The country can quickly mobilize and coordinate emergency supplies through the platform when a public emergency occurs. At the same time, different regions can also achieve efficient communication through the emergency logistics information platform to ensure the stable operation of the emergency management system.

Finally, the government needs to focus on the rational allocation and training of the social labor force. It is recommended that the government strengthen the supervision and regulation of the labor market. The labor force’s skills and emergency responsiveness can be improved through training and education policies, providing talent guarantee and support for constructing emergency logistics systems in various regions.

### 6.3 Shortcomings and prospects of this paper

This paper aims to evaluate the emergency logistics responsiveness of different regions in China and identify regional differences by analyzing the key elements of emergency logistics responsiveness. Due to time constraints and data acquisition challenges, this study still has limitations and deficiencies and needs further research. Firstly, this paper analyzes the differences in emergency logistics responsiveness among regions in China under the impact of public emergencies. However, the impact on emergency logistics responsiveness differs due to the various characteristics and scale of public emergencies. However, this paper failed to analyze this issue further. The reason is that the currently disclosed emergency-level classification indexes for public emergencies need to be further refined, which will be the focus of future research. In addition, when exploring the reasons for regional differences in emergency logistics responsiveness, this study has not yet conducted a detailed analysis of the regional differences and causes of the components of emergency logistics responsiveness. In the meantime, the differences in the variation in regional emergency logistics responsiveness components under the impact of public emergencies were not explored. This direction also needs to be explored in depth in future research.

## Data Availability

The data analyzed in this study is subject to the following licenses/restrictions: data will be made available on request. Requests to access these datasets should be directed to Heng Chen, chenhengpeerless@163.com.

## Footnotes

^1^ https://www.stats.gov.cn/sj/ndsj/

^2^ www.stats.gov.cn

^3^The western region includes Xinjiang, Qinghai, Sichuan, Xinjiang, Yunnan, Gansu, Ningxia, Guizhou, Shaanxi 9 provinces (autonomous regions); the central region includes Inner Mongolia, Heilongjiang, Shanxi, Jilin, Henan, Jiangxi, Hubei, Anhui, Guangxi, and Hunan 10 provinces (autonomous regions); the eastern region includes Tianjin, Hebei, Shanghai, Zhejiang, Beijing, Liaoning, Jiangsu, Fujian, Shandong, Hainan, Guangdong 11 provinces (cities).

^4^ https://www.wind.com.cn/portal/zh/EDB/index.html
